# Consistent effect of eating rate on food and energy intake across twenty-four *ad libitum* meals

**DOI:** 10.1017/S0007114524001478

**Published:** 2024-08-28

**Authors:** Lise A. J. Heuven, Marieke van Bruinessen, Claudia S. Tang, Markus Stieger, Marlou P. Lasschuijt, Ciarán G. Forde

**Affiliations:** 1 Division of Human Nutrition and Health, Wageningen University & Research, Wageningen, The Netherlands; 2 Food Quality and Design group, Wageningen University & Research, Wageningen, The Netherlands

**Keywords:** Eating rate, Meal texture, Food intake, Ultra-processed

## Abstract

Foods consumed at lower eating rates (ER) lead to reductions in energy intake. Previous research has shown that texture-based differences in eating rateER can reduce meal size. The effect size and consistency of these effects across a wide range of composite and complex meals differing considerably in texture and varying in meal occasion have not been reported. We determined how consistently texture-based differences in ER can influence food and energy intake across a wide variety of meals. In a crossover design, healthy participants consumed twelve breakfast and twelve lunch meals that differed in texture to produce a fast or slow ER. A breakfast group (*n* = 15) and lunch group (*n* = 15) completed twelve *ad libitum* meal sessions each (six ‘fast’ and six ‘slow’ meals), where intake was measured and behavioural video annotation was used to characterise eating behaviour. Liking did not differ significantly between fast and slow breakfasts (*P* = 0·44) or lunches (*P* = 0·76). The slow meals were consumed on average 39 % ± 9 % (breakfast) and 45 % ± 7 % (lunch) slower than the fast meals (both *P* < 0·001). Participants consumed on average 22 % ± 5 % less food (84 g) and 13 % ± 6 % less energy (71 kcal) from slow compared with fast meals (mean ± SE; *P* < 0·001). Consuming meals with a slower ER led to a reduction in food intake, where an average decrease of 20 % in ER produced an 11 % ± 1 % decrease in food intake (mean ± SE). These findings add to the growing body of evidence showing that ER can be manipulated using food texture and that this has aits consistent effect on food and energy intake across a wide variety of Hedonically equivalent meals.

A growing body of research has shown that when meals are eaten at a faster rate this leads to greater intake^(1)^, with supporting evidence from acute feeding trials^(2)^, prospective cohort studies^(3)^ and cross-sectional studies^(4,5)^. The impact of faster eating rates (ER) on energy intake is further accentuated when consuming foods with a higher energy density^(5,6)^. Eating rate (g/min) is defined as the amount of food consumed per unit time, while energy intake rate (EIR) (kcal/min) is the amount of energy consumed per unit of time. Both eating rate and EIR are contingent on an individual’s drive to eat and the structure and nutritive properties of the food being consumed^(7)^. A food’s texture can directly impact eating rate^(8–10)^, where food textures that have shorter structure breakdown during mastication, require less lubrication and take less time to form a swallowable bolus are consumed at faster eating rates^(11–13)^.

Faster eating rates have been shown to increase *ad libitum* energy intake within meals^(2,14–16)^ and consuming a fixed calorie portion at a faster rate results in a lower satiety response on a calorie for calorie basis^(17–19)^. Eating rate and EIR may also exert a sustained effect on habitual energy intakes, prompting consistently higher meal size over a longer time frame. This was shown in a recent randomised controlled inpatient feeding trial which compared energy intakes from minimally processed and ultra-processed food (UPF) diets over 14 d on each diet arm. The trial showed that the UPF diet led to a sustained average higher daily energy intake of 508 kcal/d and was linked to 0·9 kg weight gain^(20)^. Greater energy intakes on the UPF diet (increase of 144 kcal at breakfast, 248 kcal at lunch and 108 kcal at dinner) compared with a minimally processed diet were associated with a faster eating rate and almost 50 % increase in EIR. Further research is needed to better understand whether the observed differences in food and energy intake were causally attributable by meal texture-based differences in eating rate.

Numerous studies to date have explored the impact of food texture on eating rate and intake for model foods or individual food items given in fixed portions. In contrast, a limited number of studies have investigated the impact of texture on eating rate and intake in meals as they are commonly consumed, for example, realistic meals with multiple components^(15,16)^. The relationships between texture, eating rate and *ad libitum* food intake of composite mixed meals tend to be more complex, as meal components interact during consumption, influencing meal eating rate and total intake. Previous research has shown that eating rate can be reduced by an increased viscosity^(6,21–24)^, increased hardness^(2,9,25–28)^, smaller unit size^(29–34)^ and decreased initial moisture/lubrication^(22,27,28,34–38)^. While different specific texture properties of food have been shown to influence eating speed independently, combinations of texture manipulations have been shown to be most effective in slowing eating rate^(27,28)^. However, translating this from simple textures or composite foods to complex meals requires further research on the impact of meal components’ textures on eating rate. Several proof of principal studies have suggested that the effect of meal texture on eating rate could have a meaningful impact on food and energy intake within meals^(2,6,9,16)^. However, (i) the consistency of these effects across a wider range of composite and complex meals with different textures and meal occasions has not been explored yet. The majority of previous studies explored the effect of food texture on eating rate in either model foods, meal components or single meals. Consequently, (ii) relationships between texture-based reductions in meal eating rate and the associated effect size of the decrease in food intake across a broad range of complex, composite meals consumed at different meal occasions have not been established yet.

The aim of the current study was to determine the consistency of texture-based differences in eating rate (g/min) on food (g) and energy (kcal) intake across a wide variety of breakfast and lunch meals. Our hypothesis was that meals with textures that encourage a slower eating rate would consistently result in lower food and energy intakes compared with meals with textures that promote a faster eating rate.

## Materials & methods

### Study design

Two groups of participants (*n* 15/group) were each recruited to consume twelve meals (twenty-four total) in two full randomised crossover designs. The primary manipulation was the design of meals to have textures likely to facilitate a ‘slow’ or a ‘fast’ ER. One group (*n* 15) consumed breakfast (6 × fast, 6 × slow) and the other group (*n* 15) consumed lunch (6 × fast, 6 × slow). All participants (*n* 30) attended either twelve breakfast or twelve lunch sessions with one *ad libitum* meal per session in randomised order. Participants attended two sessions per week and at least 2 d between the sessions to minimise possible carryover effects from one *ad libitum* meal to another. Participants received monetary compensation upon completing the study. This study was conducted according to the guidelines laid down in the Declaration of Helsinki, and all procedures involving human participants were approved by Social Sciences Ethical Committee of Wageningen University under the Umbrella protocol (2022-118-SBSEB-prc) and were registered at Clinicaltrials.gov Clinical Trial registry: NCT05615350 (https://clinicaltrials.gov/study/NCT05615350). Written informed consent was obtained from all participants.

### Participants

A sample size calculation (G * Power version 3.1.9.4) was used to estimate that a minimum of fifteen participants was required to obtain a significant 200 kcal difference between fast and slow meals (*α* = 0·05, power 1-*β* = 0·80), based on a previous study investigating the effect of food texture on *ad libitum* food intake (soft meals 756 kcal, 95 % CI 699, 814 kcal; hard meals: 556 kcal, 95 % CI 510, 601 kcal)^(16)^. A conservative assumption was made that within-subject energy intake correlations was 0·1.

Inclusion criteria for the study were healthy, normal appetite, between 18 and 55 years old, BMI between 18·5 and 30 kg/m^2^, non-smoker, speak and understand English without difficulty, and commonly consuming three meals a day (5 d a week) around the same times. These inclusion criteria were used to have a representative sample of healthy adults. Exclusion criteria were having difficulties with eating, having braces or oral piercing, smoking, consuming on average more than twenty-one glasses of alcohol per week, not willing to stop using drugs during the study period, having allergies or intolerance to any ingredient of the test meals, not willing to eat the test food, having extensive facial hair that would interfere with video coding, following an energy-restricted diet during the last 2 months, gained or lost 5 kg of body weight over the last half year, doing intensive exercising more than 8 h per week, having a low score (≤1) for the expected liking of the individual test components on a nine-point category scale and being unfamiliar (<6 on a nine-point category scale) with more than 25 % of the test meals.

### Test meals

The study consisted of twelve breakfast meals and twelve lunch meals (twenty-four meals in total). Two meal occasions were included to have a wide range of both cold meals (during breakfast) and hot meals (during lunch), and each meal occasion was evaluated by separate participant groups to minimise participants fatigue and carryover effects between test meals^(39)^. To enable comparison with recent findings linking eating rate to energy intake for UPF meals, the ingredients of the meal components were selected for all meals that met the previously described criteria for Nova 4 (‘ultra-processed’)^(40)^. The Nova classification system consists of four groups that range from unprocessed or minimally processed foods (group 1) to UPF (group 4). Nova group 4 defines UPF as: ‘Formulations of ingredients, mostly of exclusive industrial use, that result from a series of industrial processes’^(40)^. The Nova group of each ingredient of a meal was determined independently by two coders using a pre-defined Nova definition. This approach was adopted due to the subjective nature of the Nova classification scheme which is open to misinterpretation. There was disagreement for 11 % of the breakfast ingredients and 7 % of the lunch ingredients. These ingredients were classified by a third coder to reach consensus. For breakfasts, 94 % of the meal energy was derived from ultra-processed ingredients, and this was 87 % for the lunch meals.

The selection of specific foods to ensure texture-based differences in eating for each breakfast and lunch meal was based on literature^(14,41–43)^. Meals were extensively pilot-tested where the eating rate and liking of small portions were determined with a separate panel of pilot test participants. A summary description of the breakfast and lunch meals selected and included in the trial after the extensive pilot testing is shown Table 1. Fast meals tended to have softer textures with a more lubricating consistency, whereas slow meals tended to have harder, drier, more elastic and more viscous textures. For further information on approaches used to manipulate eating rate using meal texture, see refs. 11 and 12. All meals consisted of commercially available food products purchased in supermarkets in the Netherlands. More detailed information about the meals can be found in the online Supplementary Material. For comparison purposes, breakfast and lunch meals were organised into pairs and matched for portion size (g), total energy (kcal), energy density (kcal/g), variety (number of components) and visual volume. The fast and slow meals were also matched to be overall equivalent for energy from macronutrients and fibre content served (Table 2). The meal components were served individually on plates or bowls allowing participants to self-serve their portion *ad libitum* (individual mini-buffet). Thrice the standard portion (1500 g) of each meal was served to facilitate *ad libitum* meal consumption, with additional food always made available in the event a participant completed the full portion. If a participant finished a full portion, a second full portion was offered immediately, though this occurred on only two occasions across all 360 *ad libitum* test meals. All meals were served with 120 ml of water, and the weight of each component and water were measured before and after consumption (g). Amount consumed (g) was converted to energy (kcal) by multiplying by the energy density (kcal/g) of each meal component.

**Table 1.** Description of the fast and slow matched-pairs of breakfast meals and lunch meals

Fast meals	Slow meals
*Breakfasts*	*Breakfasts*
FB1 Wrap sandwich, cake and chocolate milk	SB1 Cracker with sausage, biscuits and quark
FB2 Pancakes, croissants and smoothie	SB2 Quark with muesli, ginger bread and cookies
FB3 Egg sandwich and smoothie	SB3 Chicken cheese bagel and fruit
FB4 Peanut butter jelly sandwich and smoothie	SB4 Peanut butter bagel and quark
FB5 Chocolate cake, porridge and crepes	SB5 ‘Honey-loops’, cracker with jam and cookies
FB6 Cakes, apple sauce and smoothie	SB6 Quark with cruesli, oatmeal bars and fruit
*Lunches*	*Lunches*
FL1 Pasta bolognese, soft buns and soft salad	SL1 Spaghetti, hard buns and soft salad
FL2 Tortillas, minced meat, rice and maize	SL2 Chicken, rice, maize and chips
FL3 Mashed potato, vegetarian balls, spinach and maize mix	SL3 Baked potato, smoked chicken, beans and carrot
FL4 Croquette burger, tomato soup and soft salad	SL4 Hotdog, chips and hard salad
FL5 Hutspot (mashed potato, carrot and onion with beef stew)	SL5 Sauerkraut dish (grated potato, spiced sauerkraut and smoked sausage)
FL6 Quiche, mushroom soup and couscous salad	SL6 Tagliatelle, roasted bread and steamed vegetables

FB, fast breakfast; SB, slow breakfast; FL, fast lunch; SL, slow lunch.

**Table 2.** Average nutritional composition of the meals. Data are presented as mean ± sd

Nutritional composition	Breakfasts				Lunches			
	Fast ER		Slow ER		Fast ER		Slow ER	
	Mean	SD	Mean	SD	Mean	SD	Mean	SD
Portion size (g)	1510	10	1510	11	1514	10	1510	10
Energy (kcal)	2844	317	2846	304	2114	582	2269	580
Energy density (kcal/g)	1·88	0·21	1·89	0·21	1·40	0·38	1·50	0·38
Ultra-processed (En%)*	96	9	92	9	92	10	81	14
Carbohydrates (En%)	53	12	60	13	45	16	44	19
Mono- and disaccharides (En%)	23	10	20	9	9	4	6	5
Fat (En%)	32	8	29	9	37	27	33	13
Protein (En%)	17	16	24	18	16	5	12	6
Fibre (g/100 kcal)	1·0	0·7	1·6	0·1	1·4	0·6	1·5	0·3

ER, eating rate; En, energy.

* According to Nova 4, classified by two independent researchers.

### Procedure

Participants were recruited by social media, flyers and mailing lists from Wageningen and surroundings. Interested participants were invited to an information and screening session. They received information about the study and its goals. To conceal the true aim of the study, participants were informed that the purpose of the study was ‘to investigate the effect of emotions on eating behaviour’. On completion of the study, participants were debriefed on the real purpose of the study (no participant guessed the aim of the study correctly). Participants signed an informed consent and were asked to complete a questionnaire detailing general health and self-reported eating rate^(44)^, the Dutch Eating Behaviour Questionnaire (DEBQ) ^(45)^ and the Reasons Individuals Stop Eating Questionnaire (RISE-Q) ^(46)^. During the introductory session, participants were asked to rate their expected liking of and familiarity with all test meals and had their height and weight recorded (Seca 213 stadiometer and Seca 704 column scale). Participants’ eating rate of a 15 g piece of raw carrot (width and depth of 12·7 mm) was quantified in duplicate^(28)^.

After participants with availability for only one of the two groups (breakfast or lunch group) were allocated, the other participants were randomly allocated to one of the two meal groups. Participants were randomly allocated to a time slot and scheduled for twelve *ad libitum* test meal sessions. Participants were asked to refrain from intensive exercise and alcohol 24 h before each test session and to eat the same amount of dinner the night before each test session (not provided) between 18:00 and 20:00. To standardise appetite on the day of each test meal, participants in the breakfast group were requested not to eat or drink anything except water before the test meal breakfast. Participants in the lunch group consumed one glass of water and a fixed portion standardised breakfast which was provided by researchers and consumed at home 4 h before each lunch test session. The standardised breakfast (559 kcal) consisted of a package of multigrain breakfast biscuits (Liga Belvita: 50 g), a package of strawberry yogurt (Melkunie Breaker: 200 g), an apple (135 g) and a carton box of apple juice (AH private label: 200 ml).

Meal sessions lasted approximately 30 min and were held in the sensory booths at the Human Research Unit at Wageningen University. Breakfast sessions were between 8:00 and 9:30 am, and lunch sessions were between 12:00 and 13:30 pm. Participants consumed their meals at the same time slot for all twelve sessions. At the beginning of each session, participants rated their pre-meal appetite (hunger, fullness, thirst, desire to eat and prospective consumption) on a 100 mm anchored line scale ranging from ‘Not at all’ to ‘Extremely’. Participants then received a test meal and were asked to take a single bite and rate individual meal components on liking, familiarity, flavour intensity and three texture attributes (hardness, thickness and dryness). Hardness was defined as *‘The force needed to take a bite. An example of “Not at all” are mashed bananas and an example of “Extremely” are roasted peanuts’*. Thickness was defined as *‘The resistance of the food to flow in the mouth and is related to its viscosity. An example of “Not at all” is water and an example of “Extremely” is pudding’*. Lastly, dryness was defined as *‘The sensation of the absence of liquid/moisture in the food. An example of “Not at all” is watermelon and an example of “Extremely” are crackers’*. In addition to the sensory attributes, participants were asked to rate liking, desire to eat, familiarity and expected satiety of the entire meal after consuming one bite. All ratings were performed on 100 mm anchored line scale ranging from ‘Not at all’ to ‘Extremely’, except for familiarity which was rated on a nine-point category scale. After the ratings, participants were instructed to eat until they felt comfortably full. Participants were informed that they do not need consume foods in whole units (e.g. they could take a bite from a cookie and leave the rest) to limit unit bias. Meal consumption time was measured with a stopwatch and eating behaviour during the meal was recorded on video. At the end of each test meal, participants provided a reason for why they stopped eating (based on RISE-Q^(46)^) and rated their post-meal appetite.

### Behavioural coding of eating rate and food oral processing

Participants were video-recorded using a webcam (Logitech C310 – HD Webcam) and action camera (EKEN H9R Action-cam) positioned at face level while consuming each test meal. Participants could not see themselves while being recorded and were requested not to move their head too much while eating their meal. *Post hoc* behavioural coding was completed for a representative subset of videos to establish the average oral processing behaviours and microstructural parameters for each meal. A total of 165 (46 %; 7 participants × 24 meals except for 3 missing/incomplete videos) of the 360 videos (15 participants × 24 meals) were manually annotated across breakfast and lunch meal conditions. For these videos, oral processing behaviour was manually annotated by trained video coders using a coding scheme developed previously^(14)^ using the ELAN version 4.9.2 (Max Planck Institute for Psycholinguistics, The Language Archive). The coding scheme included four point events (number of bites, chews, sips and swallows) and three continuous events (eating duration per bite, duration per sip and total cumulative active consumption duration (all in seconds)). Breakfast and lunch meals were independently annotated by trained video coders and after coding 11 % of the videos, data were checked for agreement. Overall, the intraclass correlation coefficients (ICC) were between 0·92 and 1·00 across all oral processing behaviours for breakfasts and lunches across the coders, indicating excellent consistency (ICC > 0·90^(47)^). The remaining videos were coded separately, and the data were collated to derive summary measures of oral processing for each sample. All ICC between the coders can be found in the online Supplementary Material.

The meal eating rate (g/min) was calculated by dividing the total consumed meal weight (test food + water) by the total consumption duration measured with the stopwatch. The EIR (kcal/min) was defined as the consumed energy divided by the total consumption duration of the meal measured by stopwatch. The average bite size (g) was calculated by dividing the weight of the consumed test food by the total number of bites and sips of the test food, the number of chews per bite by dividing the number of chews by the number of bites and sips of the test food, the number of chews per gram (g^–1^) by dividing the number of chews by the consumed weight of the test food, oro-sensory exposure (OSE) time (min) by the summation of the bite and sip durations of the test food, OSEg (s/g) by dividing the OSE time by the weight of the test food and the chewing frequency (chews/s) by dividing the number of chews by the chewing time of solid foods. Moreover, eating rate of only the test foods (test food intake divided by OSE time) from the videos was subtracted. Since the eating rates obtained with the stopwatch (including water intake; of all participants) as proxy for the eating rates obtained with video coding (excluding water intake; of half of the participants) showed excellent reliability for the breakfasts (ICC = 0·98) and good reliability for the lunches (ICC = 0·86), only the eating rate obtained with the stopwatch is reported in the result section.

### Statistical analysis

Statistical analyses were performed in R version 4.1.1 (R Core Team, R Foundation for Statistical Computing). Two-tailed *P* values <0·05 were considered statistically significant. Descriptive data on sensory attributes, oral processing and appetite were analysed using paired *t* tests with the package ‘stats’. The ICC was calculated using the package ‘irr’. Analysis on the main outcomes was performed by repeated-measures linear mixed effect model using the packages ‘lme4’ and ‘lmerTest’. The models included condition (fast or slow) as fixed factor and participant as random factor. Multiple variables – including liking, test order, pre-appetite and reasons to stop eating—were investigated as possible confounders. No significant interaction effects were observed. Inclusion of liking, test order, pre-appetite and reasons to stop eating as covariate did not significantly change the outcomes of the data analysis, suggesting that these are not confounding factors of our study. Therefore, these variables (liking, test order, pre-appetite and reasons to stop eating) were not included in the final models. Separate models were made for the breakfast and lunch group. The estimation of the decrease in intake with a 20 % decrease in intake was calculated by applying a linear model to each individuals’ data. Using the intercept and slope, the percentage of decrease in intake for a 20 % decrease in eating rate was calculated, and these outcomes were than averaged across participants. One meal of one participant was excluded from this analysis since this point was an outlier for the participants’ eating rate as assessed with interquartile method (>1·5 * IQR)). Multiple factor analysis was performed on the pooled breakfast and dinner data for visual interpretation using the packages FactoMineR’ and ‘factoextra’^(48)^. Repeated-measures correlation coefficients were obtained using the package ‘rmcorr’. The β coefficients of component intake and sensory ratings of pooled data were obtained using repeated-measures linear mixed effect model with the sensory ratings as fixed factor, participant as random factor and meal as covariate (to correct for intake of components in the same meal).

## Results

### Participant characteristics

In total, thirty participants were included and all participants finished the study. The breakfast and dinner groups were similar on general characteristics and eating behaviour measures (Table 3).

**Table 3.** Participant characteristics. Data are presented as mean ± sd

	Breakfast group (*n* 15)		Lunch group (*n* 15)	
	Mean	SD	Mean	SD
Male (n)	6		2	
Age (y)	24·8	3·3	24·9	7·3
BMI (kg/m^2^)	22·5	3·2	22·7	2·6
Self-reported eating rate*	2·9	0·8	2·9	0·7
Carrot eating rate (g/min)†	20	38	20	70
Restrained eater score‡	2·0	0·7	2·3	0·7
Physical satisfaction as reason to stop eating score§	5·4	1·4	5·4	0·6

* Self-reported eating rate was measured on a scale from (1) very slow to (5) very fast^(44)^.

† The carrot eating rate (g/min) was used to characterise the observed eating rate of participants consuming a 15 g of raw carrot stick in duplicate.

‡ The restrained eater score was measured using the validated Dutch Eating Behaviour Questionnaire (DEBQ)^(45)^.

§ The physical satisfaction as reason to stop eating score was measured using the validated Reasons Individuals Stop Eating Questionnaire (RISE-Q)^(46)^.

### Oral processing, liking, sensory properties and appetite

For all fast and slow pairs of breakfast and lunch meals, the meals with textures designed to have a fast eating rate were consumed with a faster eating rate than the corresponding meals with textures designed to have a slow eating rate. Participants consumed fast breakfasts on average with a 39 % ± 9 % higher eating rate (g/min) compared with slow breakfasts and fast lunches on average with a 45 % ± 7 % higher eating rate compared with slow lunches (mean ± s e, Table 4). Fast meals were consumed on average with a 41 % ± 7 % (breakfast) and 34 % ± 10 % (lunch) higher EIR (kcal/min) compared with the slow meals at breakfast and lunch (mean ± s e). Behavioural annotation enabled a comparison of the micro-structural patterns of eating and showed that fast meals were consumed on average with larger bite sizes, fewer chews and shorter OSE than slow meals, with a similar chewing frequency for all conditions (Table 4).

**Table 4.** Microstructure of oral processing behaviour of fast and slow breakfasts and lunches. Data are presented as mean ± se

	Fast breakfasts		Slow breakfasts		*P*	Fast lunches		Slow lunches		*P*
	Mean	SE	Mean	SE		Mean	SE	Mean	SE	
Eating rate (g/min)	73	3	53	2	*<0·001*	64	2	44	1	*<0·001*
Bite size (g)*	17	1	13	1	*<0·001*	11	1	9	1	*<0·001*
Chews per bite*	16	1	20	1	*0·02*	12	1	14	1	0·05
Number of chews (g^–1^)*	1·1	0·1	1·7	0·1	*<0·001*	1·2	0·1	1·8	0·2	*<0·001*
Chewing frequency (chews/s)*	1·4	0·0	1·5	0·0	0·11	1·6	0·1	1·5	0·0	0·50
OSE time (min)*	6·1	0·4	8·2	0·4	*<0·001*	7·0	0·4	9·2	0·5	*<0·001*
OSEg (s/g)*	1·0	0·1	1·3	0·1	*<0·01*	1·0	0·1	1·5	0·1	*<0·001*
Energy intake (kcal)	773	25	669	23	*<0·001*	660	29	621	26	0·28
Meal intake (g)	493	18	409	14	*<0·001*	485	19	402	14	*<0·001*
EIR (kcal/min)	98	4	69	2	*<0·001*	69	3	52	2	*<0·001*

OSE, oro-sensory exposure; EIR, energy intake rate.

* For subset of participants. Assessed with video coding with fast breakfasts *n* 42 videos; slow breakfasts meals *n* 42, fast lunches *n* 40 and slow lunches *n* 41.

Almost all meals (22 out of 24; 92 %) had an average liking rating >50. Average liking and familiarity for the breakfasts and lunch meals were not significantly different between the fast and slow meals (all *P* > 0·44, Table 5). Comparison of the sensory ratings showed that the slow meal components were on average perceived as harder, dryer and thicker compared with the fast meal components (Table 5). Pre-meal appetite ratings confirmed that participants had equivalent appetite need states before each of the *ad libitum* meals. Post-meal appetite ratings confirmed that participants felt comfortably full following each meal with no differences in post-meal appetite between fast and slow breakfasts or fast and slow lunches (all *P* > 0·05). For the reason to stop eating, participants selected ‘I was full’ 60 % of the times, ‘The food is no longer appealing to me’ 13 % and ‘I was bored with the flavour’ 8 %. Appetite ratings and the reasons of the participants to stop eating are summarised in the online Supplementary material.

**Table 5.** Sensory and hedonic ratings of the meal overall and of the meal components of all participants. Data are presented as mean ± se

	Fast breakfasts		Slow breakfasts		*P*	Fast lunches		Slow lunches		*P*
	Mean	SE	Mean	SE		Mean	SE	Mean	SE	
*Meal overall*										
Liking	64	2	65	2	0·44	59	2	59	2	0·76
Familiarity	2·6	0·2	2·6	0·2	0·96	3·4	0·2	3·3	0·2	0·53
Desire to eat	65	2	66	2	0·70	60	2	62	2	0·47
Expected satiety	68	2	73	2	0·06	64	2	65	2	0·92
*Meal components* *										
Liking	65	1	68	1	0·09	57	1	61	1	*0·01*
Familiarity	2·6	0·1	2·5	0·1	0·41	3·2	0·1	2·6	0·1	*<0·001*
Flavour intensity	64	1	61	1	*0·04*	50	1	52	1	0·20
Hardness	15	1	44	2	*<0·001*	21	1	42	2	*<0·001*
Dryness	27	2	44	2	*<0·001*	30	2	41	2	*<0·001*
Thickness†	47	2	62	3		32	3	NA		

* There were in total sixteen breakfast components and eighteen lunch components.

† Only for semi-solids and liquid components of the fast breakfasts (*n* 7), slow breakfasts (*n* 5), fast lunches (*n* 5) and slow lunches (*n* 0).

### Food and energy intake

**Fig. 1.**
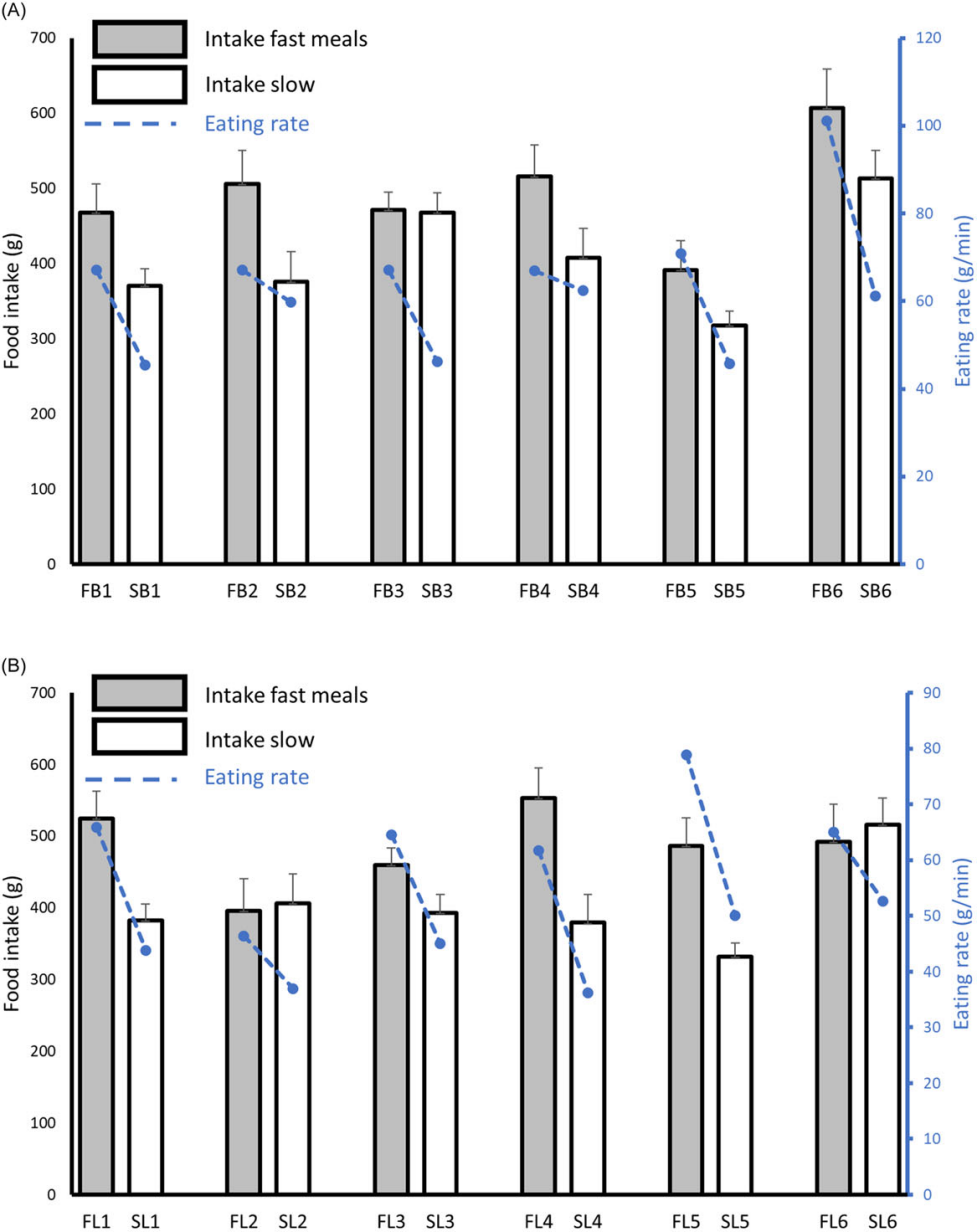
Food intake (g) and eating rate (g/min) of the fast (*n* 6 meals) and slow (*n* 6 meals) breakfast meals (A) and fast (*n* 6 meals) and slow (*n* 6 meals) lunch meals (B) of all participants. FB, fast breakfast. SB, slow breakfast. FL, fast lunch. SL, slow lunch. Mean ± se.

**Fig. 2.**
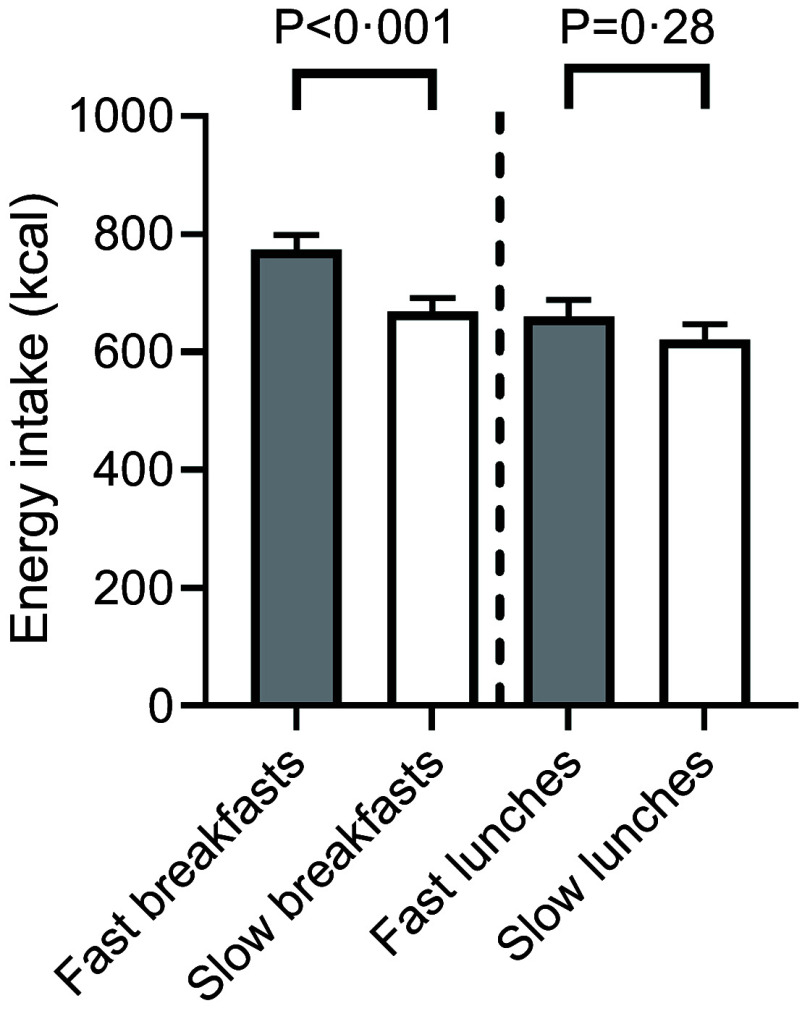
The energy intakes of the fast (*n* 6 meals) and slow (*n* 6 meals) breakfasts and the fast (*n* 6 meals) and slow (*n* 6 meals) lunch meals of all participants. The bars represent the mean ± se.

Food intake was on average 21 % ± 3 % (84 g; *P* < 0·001) higher when participants consumed the fast breakfasts compared with the slow breakfasts and 23 % ± 9 % (85 g; *P* < 0·001) higher for the fast lunches compared with the slow lunches (mean ± s e; Fig. 1). Energy intake was on average 16 % ± 8 % (104 kcal; *P* < 0·001) higher when consuming the fast breakfasts compared with the slow breakfasts (mean ± s e). Energy intake was similar (39 kcal difference; *P* = 0·28) between fast and slow lunches (Fig. 2).

The relative differences in energy intake between the fast and slow meals (16 % for breakfasts and 6 % for lunch) were smaller than the relative differences in food intake (22 %), even though the served meals had similar energy densities (Table 2). The energy densities of the breakfast meals consumed was not different (*P* = 0·28) between the fast (1·65 ± 0·05 kcal/g) and slow breakfasts (1·70 ± 0·05 kcal/g). However, within the lunch meals the energy density consumed was 0·18 kcal/g higher (*P* < 0·001) for the slow lunches (1·57 ± 0·05 kcal/g) than for the fast lunches (1·39 ± 0·04 kcal/g). This difference in *ad libitum* energy density (kcal/g) consumed between the fast and slow meals resulted in a smaller net average difference in energy consumed (kcal) than amount consumed (g).

### Consistency of the effect of eating rate on food intake

**Fig. 3.**
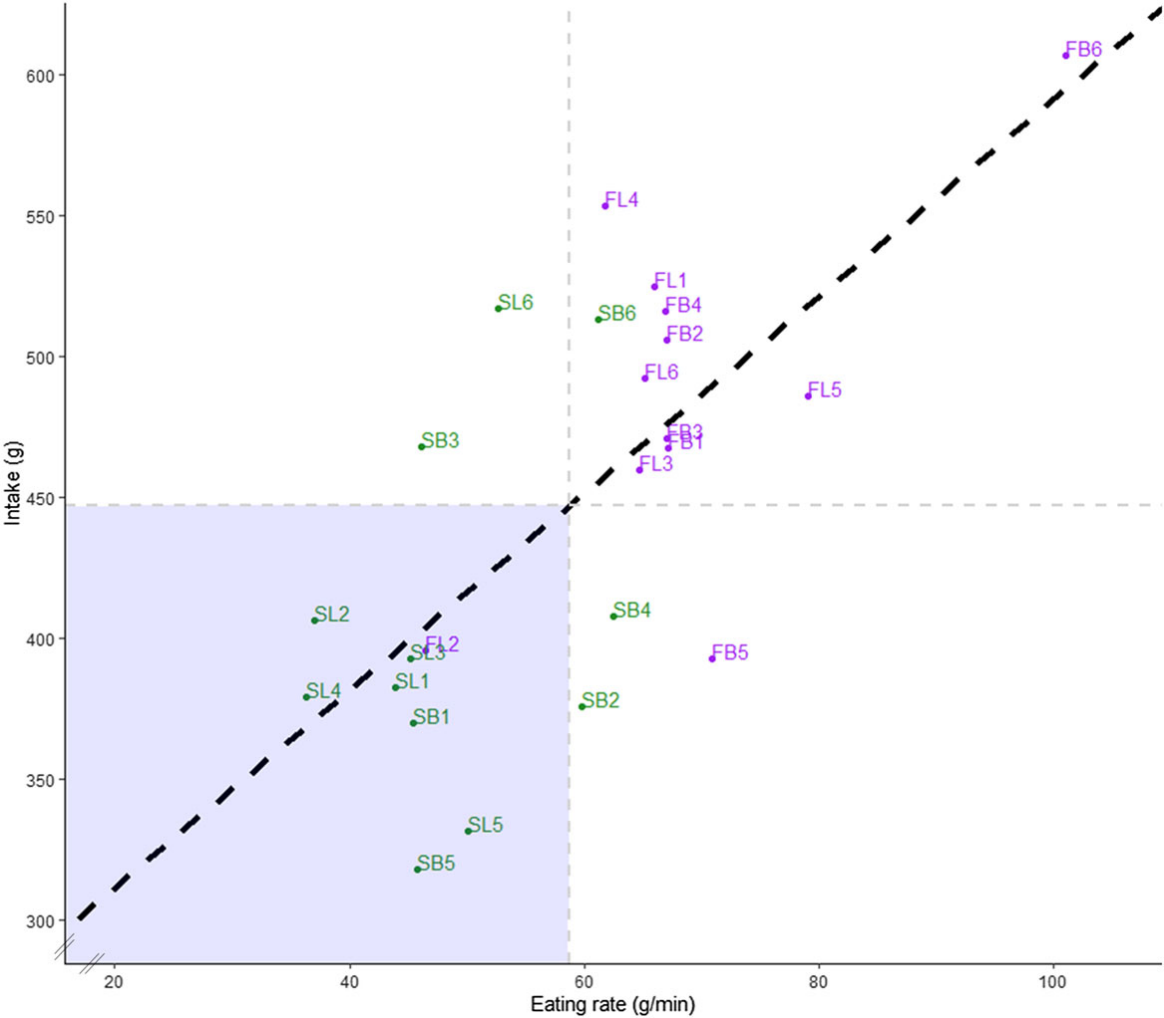
Plot of the average eating rates and intakes of the fast breakfasts (FB; purple), slow breakfasts (SB; green), fast lunches (FL; purple) and slow lunches (SL; green) of all participants. The black dashed line represent the regression line of best fit based on average values. The grey dashed lines indicate the average eating rate (59 g/min) and average intake (447 kcal) of all meals.

For ten of the twelve meal pairs (83 %), meals with textures leading to faster eating rates had a higher food intake compared with meals with textures leading to slower eating rates, though intake varied considerably between meal pairs (Fig. 1). Eating rate had a consistent effect on food intake (g) across all twenty-four meals (twelve breakfasts and twelve lunches) (Fig. 3). Eating rate was a good predictor of food intake for breakfast (*β* = 3·7, *P* < 0·001), lunch (*β* = 4·0, *P* < 0·001) and all meals together (*β* = 3·8, *P* < 0·001). Based on the average eating rate across all twenty-four meals (63 g/min for breakfast and 54 g/min for dinner), we estimate that a decrease of 20 % in eating rate resulted in a decrease of 10 % ± 1 % (lunch; range of 4–21 %) to 12 % ± 1 % (breakfast; range of 5–20 %) in food intake (mean ± s e).

### Drivers of meal intake

**Fig. 4.**
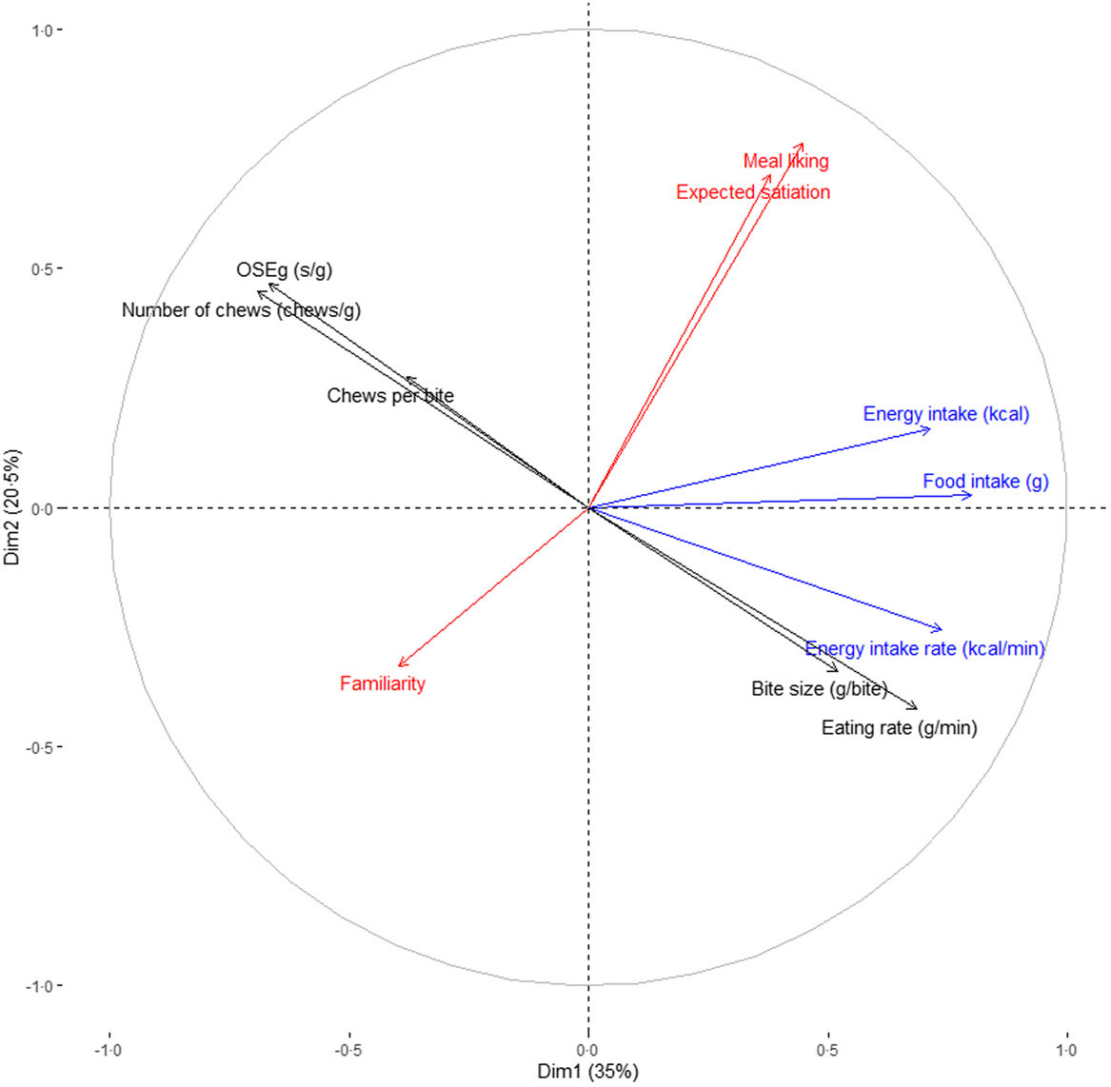
Correlation circle of multiple factor analysis performed on intake (blue; of all participants), eating behaviour (black; eating rate of all participants, other behaviours of subset of participants), liking familiarity and expected satiation (red; of all participants). The first two dimensions explain together 56 % of the variance. OSE, oro-sensory exposure.

To summarise the relative influence of the main oral processing and hedonic variables on meal intake, factor analysis was performed (Fig. 4). Figure 4 shows that food intake, energy intake and EIR of a meal were positively correlated with eating rate and bite size and negatively correlated with number of chews, OSEg and chews per bite. Participants’ ratings for meal liking and familiarity were not correlated with eating rate, EIR or any of the oral processing parameters. Repeated-measures correlation coefficients showed that food intake of the whole meal had the strongest significant correlation with eating rate (Table 6).

**Table 6.** Repeated-measures correlation coefficients of food intake (g)

	*r*	*P*
*Eating behaviour*		
Eating rate (g/min)	0·59	*<0·001*
Bite size (g)*	0·45	*<0·001*
Chews per bite (-)*	–0·33	*<0·001*
Number of chews (g^–1^)*	–0·49	*<0·001*
OSEg (s/g)*	–0·48	*<0·001*
*Liking, familiarity, expected satiation*		
Liking	0·40	*<0·001*
Familiarity	–0·20	*<0·001*
Expected satiation	0·22	*<0·001*

OSE, oro-sensory exposure.

* Assessed from video coding for half of the participants.

Variation in intake of individual meal components was primarily driven by liking (*β* = 1·71, *P* < 0·001), followed by the sensory attributes *dryness* (*β* = −1·41, *P* < 0·001) and *hardness* (*β* = −1·01, *P* < 0·001). A summary of this analysis can be found in the online Supplementary Material.

## Discussion

This study demonstrates that eating rate can be moderated in a consistent manner using hedonically appealing food textures to slow down eating rate and reduce food intake across a wide variety of representative everyday multiple-component meals. Texture-based differences in eating rate resulted on average in a 21 % difference in food intake for both breakfast and lunch meals. Based on our findings, we estimate a texture-based reduction in eating rate of 20 % will likely generate an average 10–12 % decrease in meal intake, though the size of this effect will vary from meal to meal.

Previous research has suggested that a 20 % reduction in texture-based eating rate produces a 10–15 % reduction in energy intake^(49)^, though this estimate was based on the observed differences across a much smaller set of one component *ad libitum* test meals^(12)^. The current study shows the impact of a variety of different food texture manipulations had a consistent effect on eating rate and intake across a large variety of representative meals with different consumption contexts (breakfast and lunch). Previous research has shown that children’s eating behaviours were consistently linked to food and energy intake across four *ad libitum* meals varying in composition, texture and portion size (chicken nuggets, macaroni, grapes and broccoli)^(50)^. Similarly, when the same foods are served on four consecutive weeks to adults in a similar need state, eating rate and *ad libitum* energy intake were consistent at an individual level. That study pooled data from four experimental studies using rice-based meals and demonstrated such strong behavioural consistency that energy intake on week four could be predicted by an individual’s eating rate in week 1^(51)^. When combined with the findings from the current trial, this suggests that texture-based differences in eating rate could be used to reduce eating speed and food intake in a consistent manner across a wide range of similarly hedonically appealing everyday meals.

A goal of the current study was to identify patterns of food texture combinations that consistently slow eating rate for breakfast and lunchtime meals. Many slow meals had higher perceived *hardness* and *dryness* compared with the fast meals, in line with previous research^(2,9,25–28)^. Lower moisture content and lubrication^(22,27,28,34–38)^ alongside changes in chewy and textures^(12)^ have also been shown to reduce eating speed. The study confirmed that combinations of texture manipulations seems to have the strongest effect in reducing eating rate^(27,28)^. An example of such a meal pair was ‘hutspot’ (FL5; mashed potato, carrot and onion with beef stew) which had a soft and moist texture and higher eating rate and intake compared with its slow counterpart ‘sauerkraut dish’ (SL 5; grated potato, spiced sauerkraut and smoked sausage) which had a hard, chewy and dry texture. Most of the meals were within the positive hedonic range, where liking was on average equivalent for the fast and slow versions of the paired meals. However, within the current trial, in a minority of cases (two out of twelve meal pairs), the hedonic appeal of the slow meal dominated intake behaviour, and although participants ate the meal slower, they ate more in response to the foods appeal. This highlights that hedonic aspects of a meal may surpass the effect of texture. The study aimed to reduce the influence of liking on food intake, but there might yet be residual influence from individual differences in liking that were not accounted for in our models. Although texture-based reductions in eating rate leads to decreases in food intake, it is important to acknowledge that eating behaviour is a multi-factorial response to the integrated sensory, structural and physical–chemical properties of the foods being consumed^(52)^. In addition, other individual and environmental factors such as stress, distraction, smell, sound and temperature have been shown to influence intake^(52–54)^. The relationship between food texture, eating rate and intake is complex and merit further research, though findings from the current study suggest there is consistency in how consumers translate the differences in food texture into slower eating rates that affect food intake.

The significant effect of eating rate on food and energy intake found in this study is in line with recent findings that have sought to investigate the effect of meal texture on food intake for foods that would be classified as minimally or ultra-processed. Teo and colleagues showed that food texture moderates intake across both minimally or ultra-processed *ad libitum* test meals where the slower meals were consumed with on average 21 % less food (g) and 26 % less energy^(16)^ compared with fast meals. Similarly, Lasschuijt and colleagues compared *ad libitum* intake for breakfast, lunch, dinner and dessert across a day of minimally or ultra-processed slow and fast meals. The faster meals were consumed at faster eating rate (46 %) and led to a 14 % increase in amount consumed (g) and 33 % higher energy intake compared with slower meals^(15)^. In both cases, there was no influence of degree of food processing on eating rate, amount or energy consumed and the texture-based differences in eating rate determined intake behaviour^(15,16)^. These studies highlight the possibility that higher energy intakes reported for UPF could be in part be mediated by the texture-based eating rates of the meals tested^(20,55)^. Whereas food intake results of the current study are in line with previous findings, the overall net effect on energy intake was smaller than expected, especially for lunchtime meals. This may be a result of the meals used in this study, where several components were combined to represent realistic multi-component meals. Energy density was matched at the level of the meal offered *ad libitum*, though the constituent meal components differed in energy densities, creating opportunities in the *ad libitum* paradigm for participants to select the components that they wanted to consume. An artefact of this approach was the selection and intake of some of the more energy-dense meal components within the slow meals at lunchtime. This resulted in differences in the energy density of the meal consumed and a compression of the differences in the net energy (kcal) consumed when compared with observed differences in amount (g). A similar trend was observed in the previous RCT to test the impact of minimally *vs* ultra-processed diets, where energy density consumed was significantly higher for the UPF diet than for the minimally processed diet^(20)^. Taken together, these findings highlight that foods higher in energy can have a strong impact on energy intake when consumed quickly, but there is an opportunity to accentuate the observed reduction in energy intake in slower textured foods by also reducing the energy density of these foods. Future research is needed to investigate synergies between the combinations of energy density and eating rate reductions to better control meal size and energy intake.

Limitations of the current study were the small sample size for descriptive and hedonic ratings and the controlled eating behaviour research unit environment. The ecological validity of the study should be explored in the future using controlled dietary interventions studies at the home of participants. A strength of the current study was the wide variety of commonly consumed meals that were matched for energy density, energy from macro-nutrients, liking, variety and volume. The current study used composite meals rather than individual food items or homogenous foods, which made the approach more complex but also increased the external validity of the findings. These findings will help to better understand how natural variations in the texture and eating rate of commonly consumed meals can influence habitual energy intakes from meals comprising components that are defined as ultra-processed. In line with recent studies that have investigated the effect of meal texture on eating rate and energy intake from UPF meals^(15,16)^, the current findings support the suggestion that greater energy intakes from diets dominated by UPF foods are likely to be at least partially explained by faster eating rates and higher energy density^(20)^. Whether texture-based differences in meal eating rate can lead to sustained changes in food and energy intake over a longer time period and across different populations and cultural groups remains to be seen. For example, faster eaters tend to have a higher BMI and higher risk of cardiometabolic diseases compared with slower eaters as evidenced by research results from Japan, Korea, Singapore and the Netherlands^(5,42,56–58)^. Whether the eating behaviour of populations with different BMI respond similar to changes of a foods’ texture is not clear^(56,57)^. If the effect of texture on eating rate is consistent and sustained for different populations, the effect of reducing eating rate using food texture creates an opportunity to steer behaviours that reduce the risk of excessive energy intakes.

In conclusion, our research highlights that meal texture has a consistent effect on eating rate and energy intake across a wide variety of hedonically equivalent breakfast and lunchtime meals. The consumption of meals with a slower eating rate consistently reduced the intake for both breakfast and lunchtime meals and was associated with smaller average bite sizes, greater chews per bite and longer OSE time. Future research should seek to investigate whether the acute effects observed on texture-based reductions in eating rate and intake can persist over a longer time period. These insights offer new opportunities to develop food-based strategies to either promote or limit intake of target foods through a combination of nutritive and sensory–behavioural approaches.

## Supporting information

Heuven et al. supplementary material Heuven et al. supplementary material

## Supplementary material

For supplementary material/s referred to in this article, please visit https://doi.org/10.1017/S0007114524001478
