# Supplementary material for: Consistent effect of eating rate on food and energy intake across twenty-four ad libitum meals
Source: Br J Nutr. 2024 Sep 16;132(4):535–46. doi: 10.1017/S0007114524001478 (PMC11499084; doi:10.1017/S0007114524001478)
Supplement: Heuven et al. supplementary material [file S0007114524001478sup001.docx]

# **Consistent effect of eating rate on food and energy intake**

# **across 24 *ad libitum* meals**

Lise A.J. Heuven^a,b^, Marieke van Bruinessen^a^, Claudia S. Tang^a^, Markus Stieger^a,b^,

Marlou P. Lasschuijt^a^, Ciarán G. Forde^a^

1. Division of Human Nutrition and Health, Wageningen University & Research, P.O. Box 17, 6700 AA, Wageningen, The Netherlands
2. Food Quality and Design group, Wageningen University & Research, P.O. Box 17, 6700 AA, Wageningen, The Netherlands

**Table 1.** Ingredients and pictures of the breakfast meals.

| **Fast Breakfasts** | **Slow breakfasts** |
| --- | --- |
| *1. Wrap sandwich, cake & chocolate milk*   - 229 g Wraps (AH private label) with herb cream cheese (AH private label) and chicken fillet (AH private label); - 222 g Cake roll hazelnut (Balconi); - 456 g Chocolate milk (Chocomel).   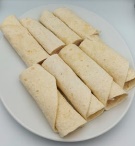 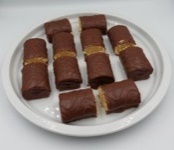 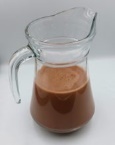 | *7. Cracker with saveloy, biscuits & quark*   - 495 g Knackebröd (AH private label) with cream cheese (AH private label), cucumber, and saveloy (Stegeman); - 177 g yoghurt-strawberry biscuits (Liga Belvita); - 840 g Protein stracciatella quark (Lindahls).   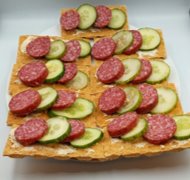 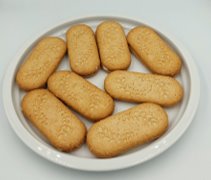 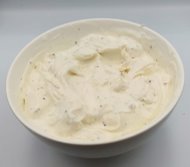 |
| *2. Pancakes, croissants & smoothie*   - 425 g Banana pancakes (AH private label) with caramel syrup (Van Gilse) - 234 g Mini croissants (AH private label) - 850 g raspberry, blueberry smoothie with grains and dairy (Vifit)   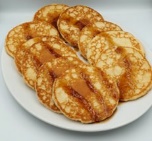 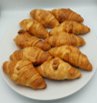 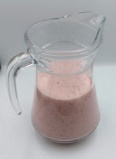 | *8. Quark with muesli, ginger bread & cookies*   - 1050 grams skimmed stracciatella quark (Optimel) with fruits and nuts muesli (Holi); - 300 g Spiced ginger bread (Peijnenburg); - 160 g Mini Bastogne cookies (Lu).   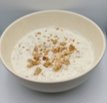 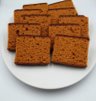 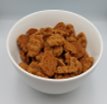 |
| *3. Egg sandwich & smoothie*   - 900 g Whole wheat sandwich (AH private label) with ham (AH private label), egg salad (Johma) and lettuce; - 500 g Strawberry, kiwi and banana smoothie with grians and dairy (Vifit)   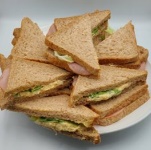 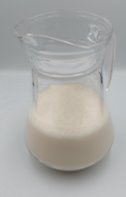 | *9. Chicken cheese bagel & fruit*   - 866 g Sesame bagel (AH private label) with grilled chicken fillet (AH private label), 20+ cheese (Milner) and rucola; - 640 g Pineapple, mango, apple and grape fruit mix (AH private label).   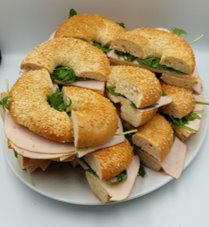 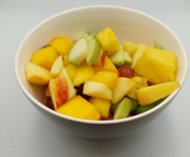 |
| *4. Peanut butter jelly sandwich & smoothie*   - 900 g Whole-wheat sandwich (AH private label) with creamy peanut butter (skippy) and strawberry jam (AH private label); - 600 g Orange and strawberry smoothie with grains (Coolbest)   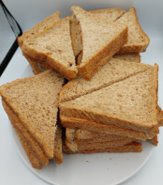 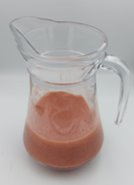 | *10. Peanut butter bagel & quark*   - 632 g Sesame bagel (Ah private label) with creamy peanut butter (skippy); - 870 g Skimmed strawberry quark (Optimel).   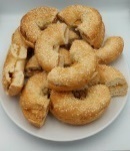 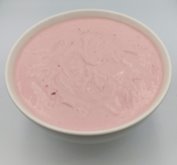 |
| *5. Chocolate cake, porridge & crepes*   - 615 g Cinnamon oatmeal porridge (Quacker) with oatmilk (Oatly); - 578 g Dutch pancakes (Jan) with strawberry jam (AH private label); - Chocolate cakes (Milka).   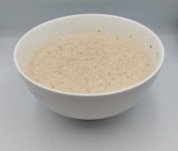 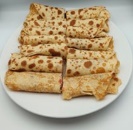 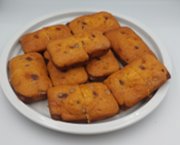 | *11. Honeyloop, rusks with jam & cookies*   - 885 g Honeyloops (Kellogs) with soymilk (AH private label); - 420 g Rusks (AH private label) with light margarine (Becel) and strawberry fruit spread (AH private label); - 225g Chocolate chip cookies (AH private label).   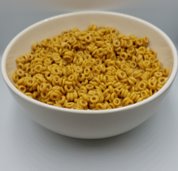 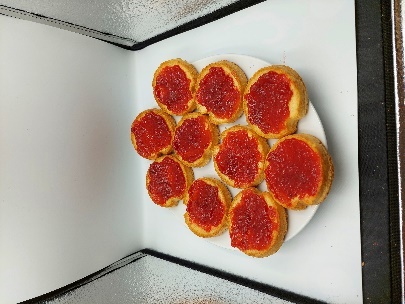 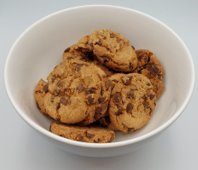 |
| *6. Cakes, apple sauce & smoothie*   - 480 g Cupcakes (AH private label); - 450 g zero sugar cinnamon apple sauce (AH private label); - 600 g Forest fruit smoothie with grains (Coolbest)   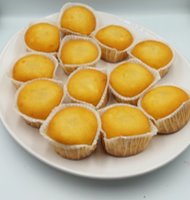 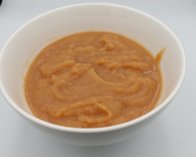 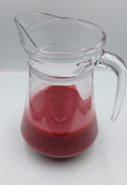 | *12. Quark with cruesli, oatmeal bar and fruitmix*   - 750 g Skimmed vanilla quark (Optimel) with chocolate cruesli (Quacker); - 280 g Oatmeal bars (Bolletje); - 500 g Pineapple, mango, apple and grape fruit mix (AH private label).   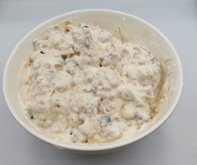 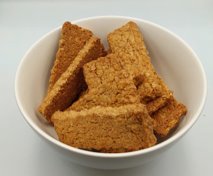 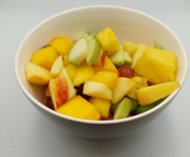 |

Table 2. Ingredients and pictures of the lunch meals.

| **Fast lunches** | **Slow lunches** |
| --- | --- |
| *1. Pasta bolognese, soft buns & soft salad*   - 850 g Macaroni bolognese (Huuskes private label) - 340 g White buns (AH private label) with herbal butter (Ah private label) - 325 g Lettuce, tomato and naturel zero dressing (Remia)   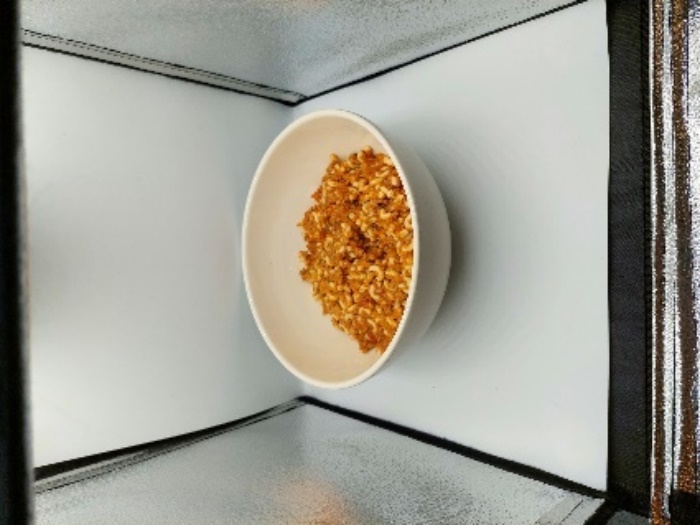 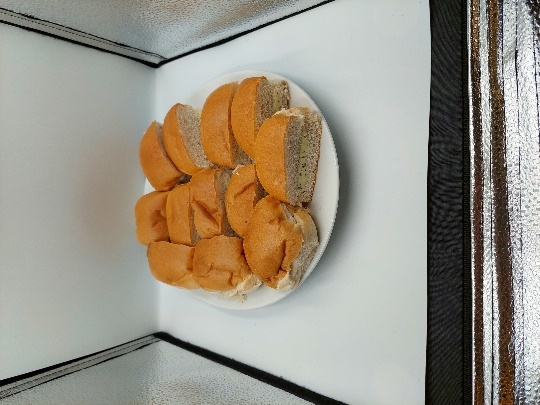 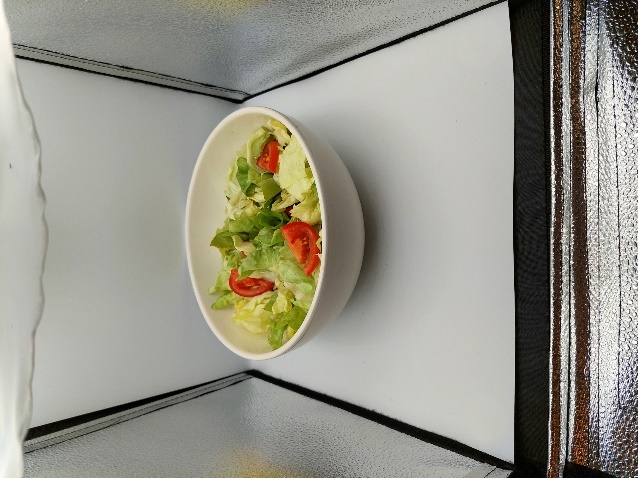 | *7. Spaghetti, hard buns & soft salad*   - 840 g Spaghetti with vegetables (Huuskes private label) - 390 g Hard buns (AH private label) with herbal butter (AH private label) - 310 g Bell pepper, cucumber, carrot julienne and naturel zero dressing (Remia)   *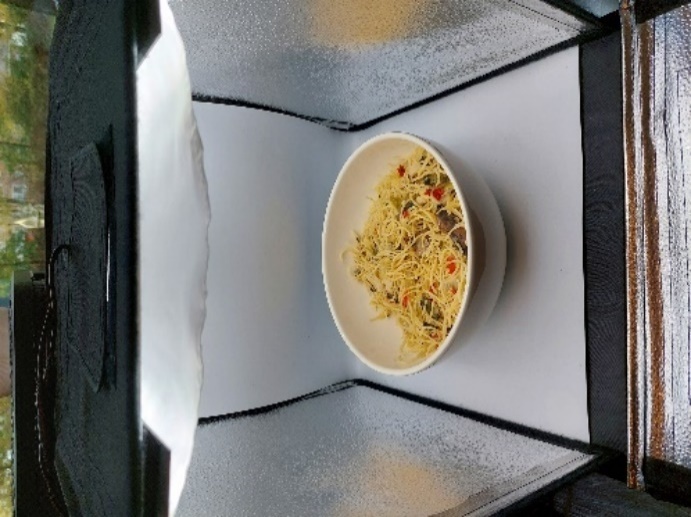 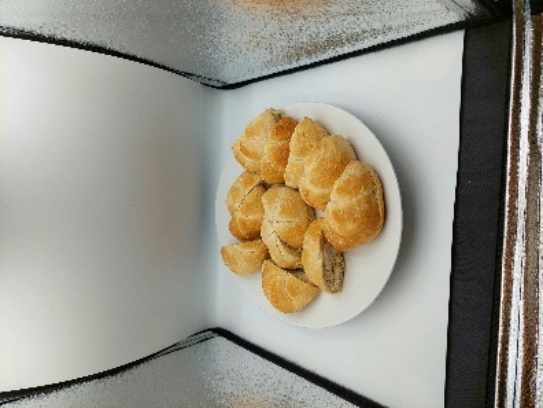 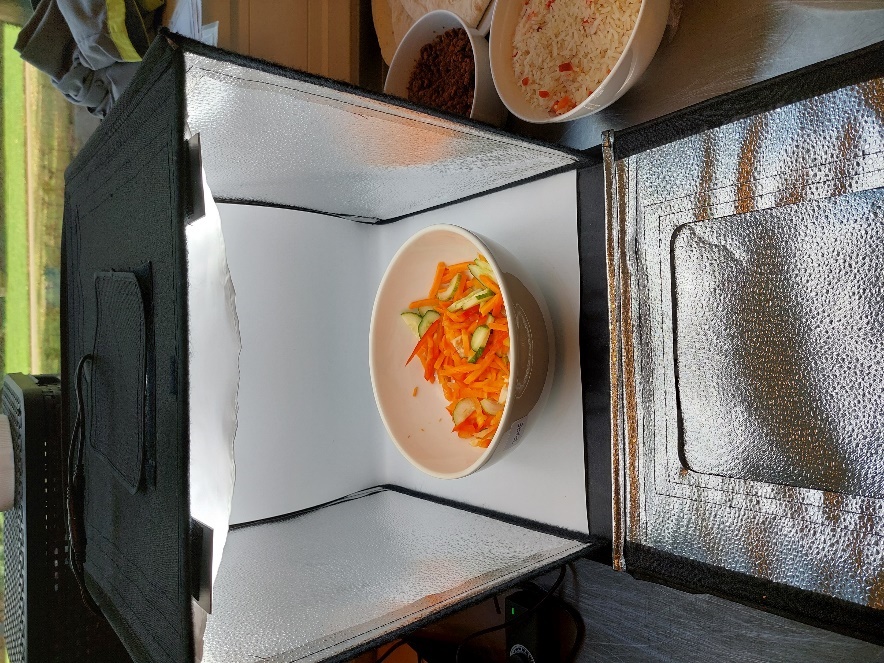* |
| *2. Tortillas, minced meat, rice & corn*   - 350 g Vegan minced meat (AH private label) - 420 g Garni rice (Huuskes private label) - 265 g Corn mix (Bonduelle) - 372 g Tortilla wrap (AH private label) - 100 g Tomato ketchup (Heinz)   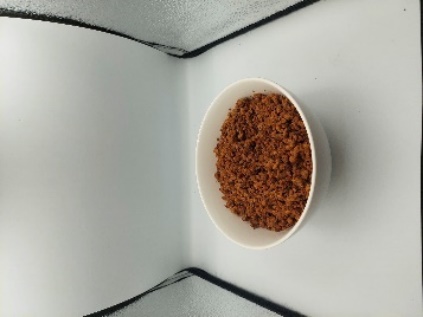 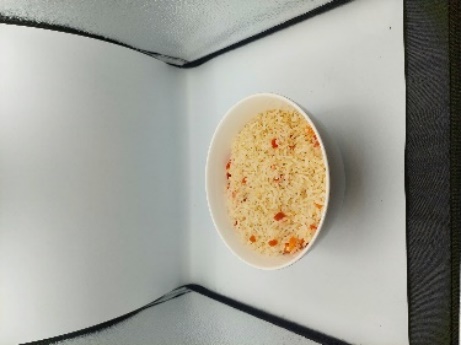 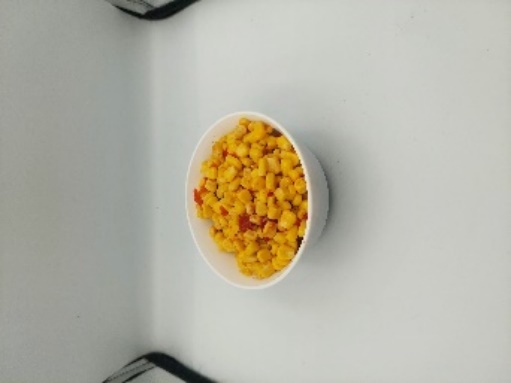 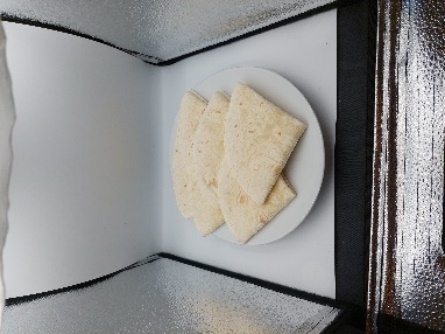 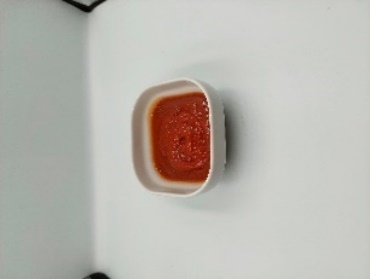 | *8. Chicken, rice, corn & chips*   - 258 g Chicken drumsticks (AH private label) - 350 g Nut rice (Lassie) - 750 g Corn cob (AH private label) - 150 g Nacho cheese chips (Doritos) - 20 g Chunky salsa (Santa Maria   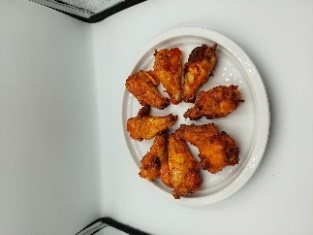 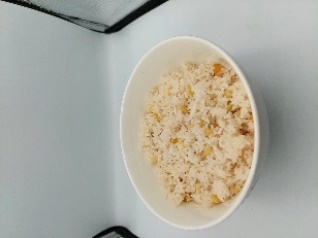 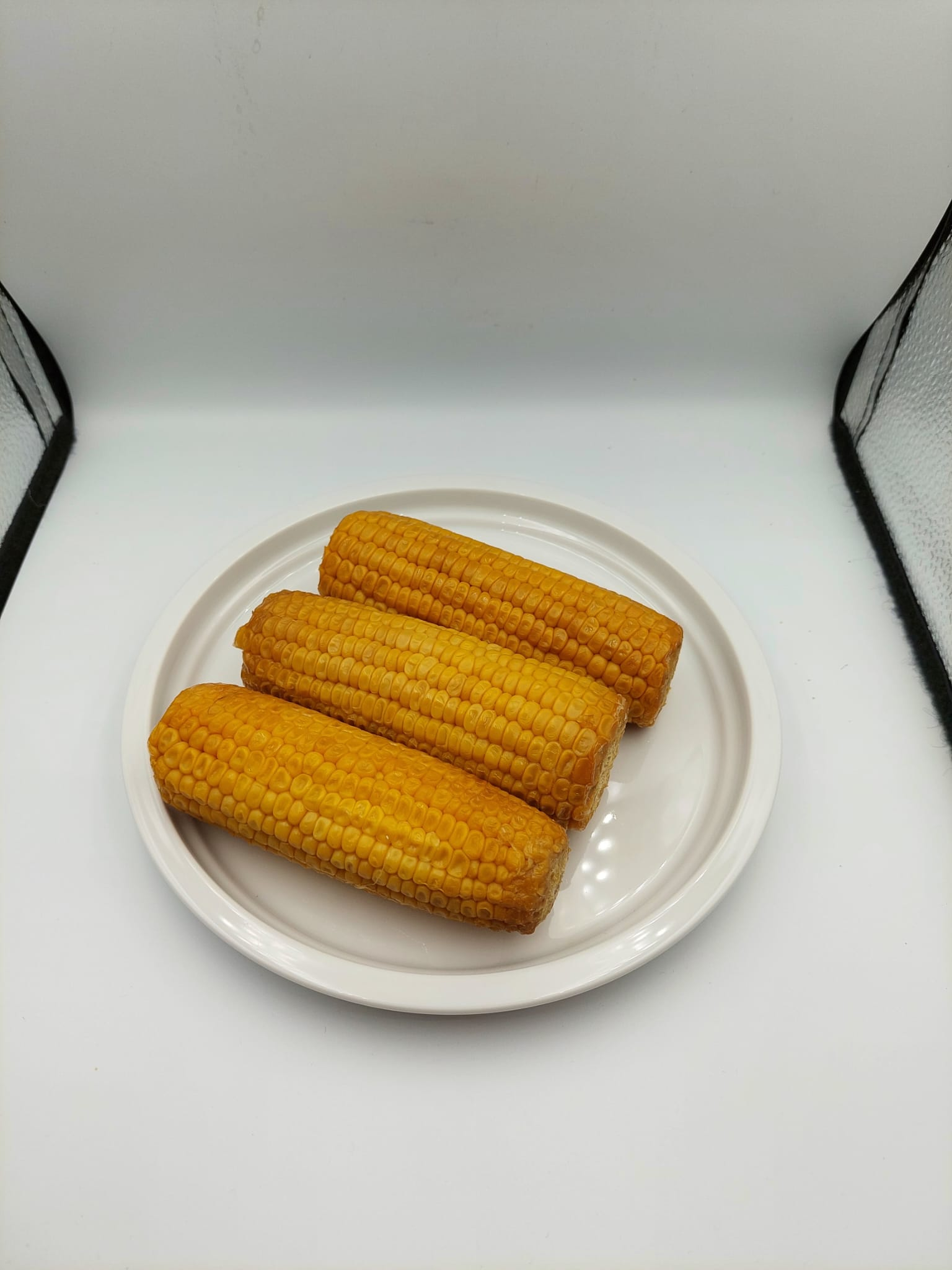 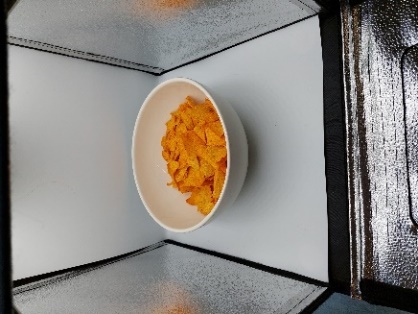 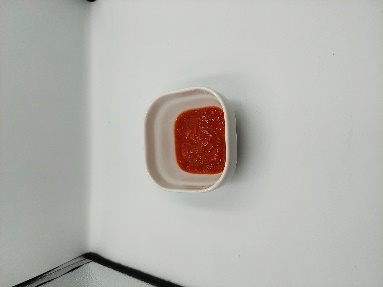 |
| *3. Mashed potato, vegetarian balls, spinach & corn mix*   - 500 g Mashed potato (AH private label) with Italian hebrs (Euroma) - 300 g Vegetarian balls (AH private label) - 425 g Spinach a la crème (Iglo) - 300 g Corn mexicana mix (Bonduelle)   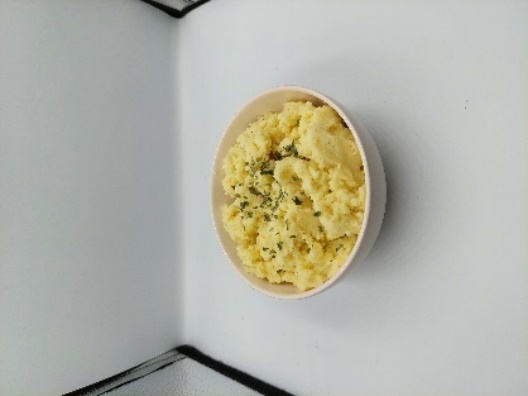 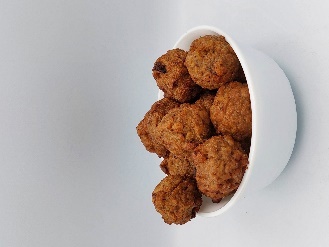 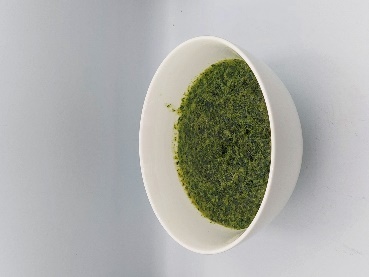 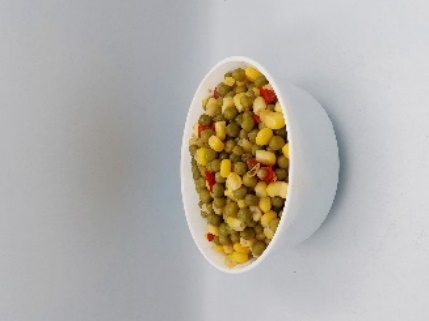 | *9. Baked potato, steamed chicken, beans and carrot*   - 369 g Baked potatoes with garlic and rosemary (AH private label) - 390 g Smoked chicken fillet (AH private label) - 429 g Green beans with tandoori sauce (Patak’s) - 335 g Carrot with twandoori sauce (Patak’s)   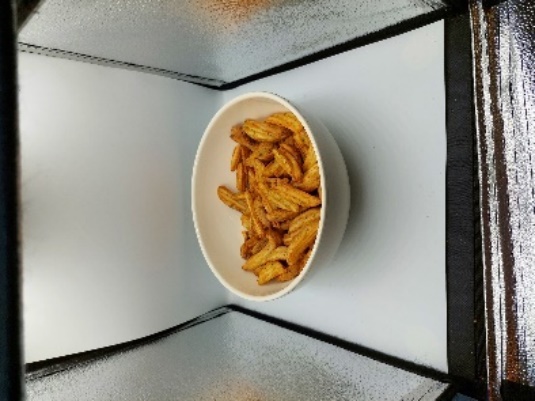 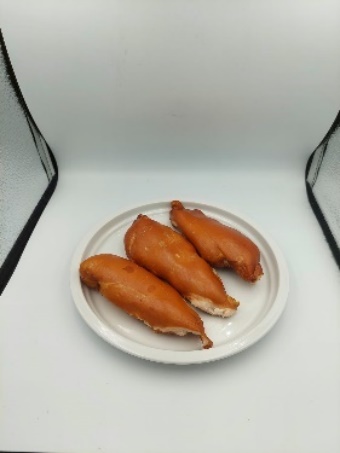 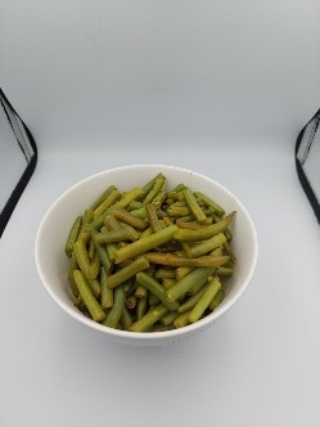 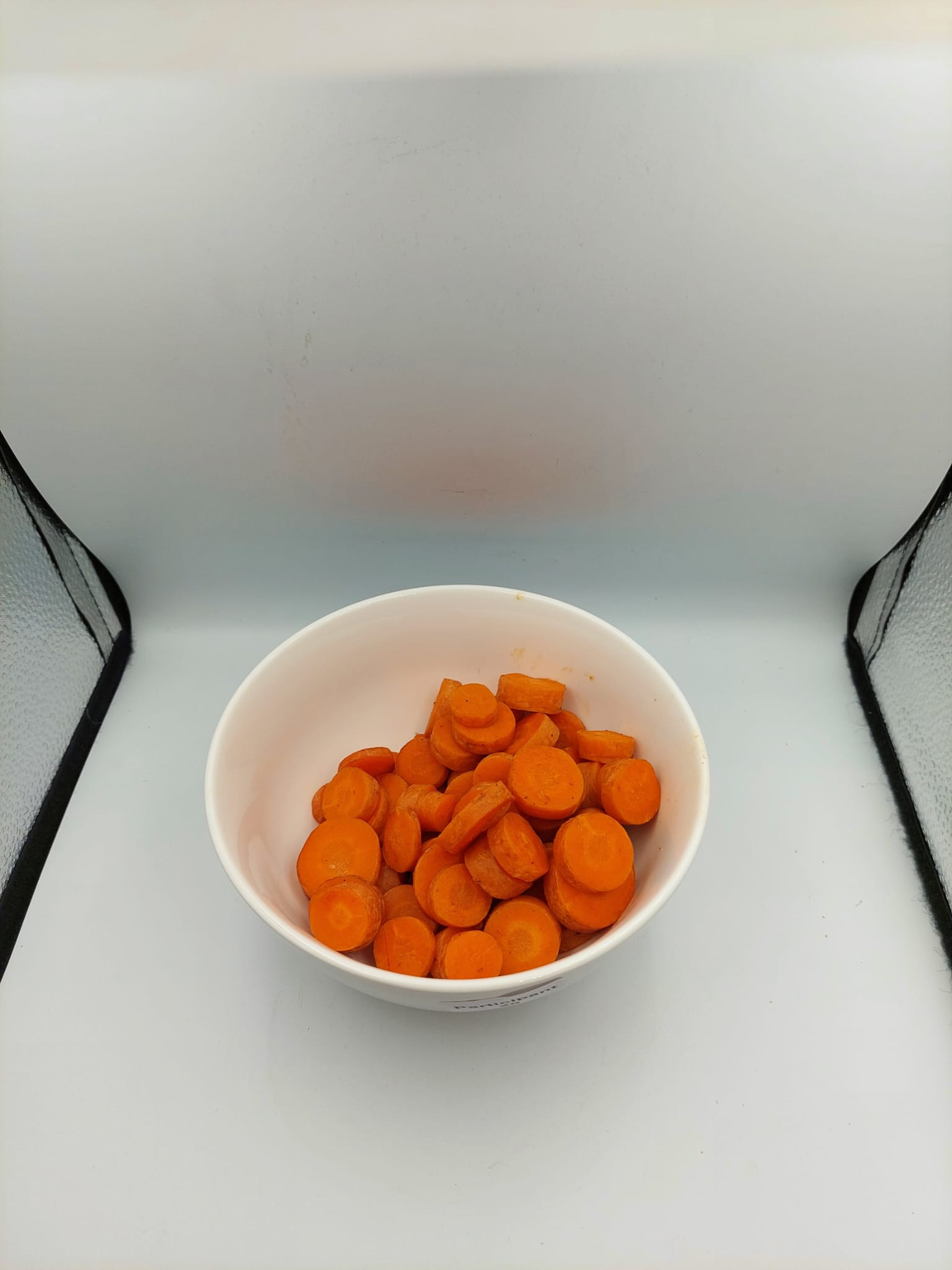 |
| *4. Kroket burger, tomato soup & soft salad*   - 592 g Kroket burger (Mora) - 639 g Spiced tomato soup (Unox) with liquid backing butter (Becel) - 280 g Lettuce, tomato and naturel zero dressing (Remia)   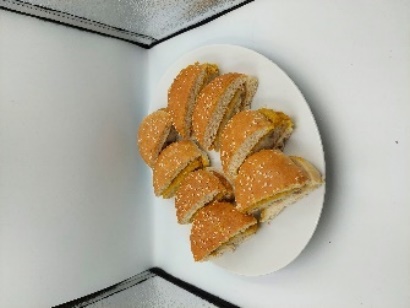 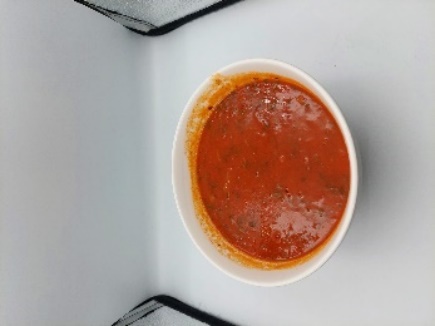 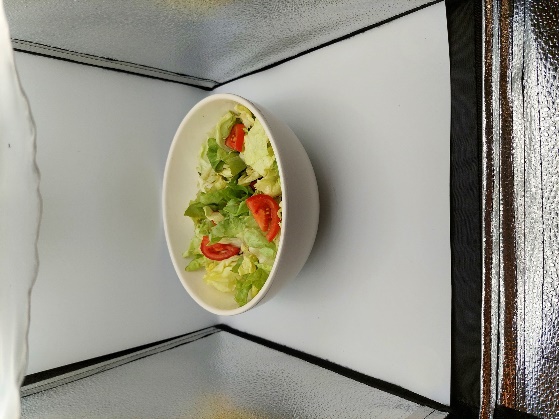 | *10. Hotdogs, chips & hard salad*   - 1038 g Schnitt rolls (AH private label) with extra lean smoked sausage (Unox) and zero tomato ketchup (Heinz) - 200 g Oven baked naturel chips (Lay’s) - 465 g Bell pepper, cucumber, carrot julienne and naturel zero dressing (Remia)   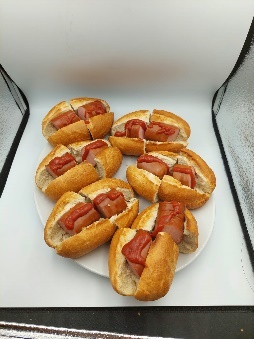 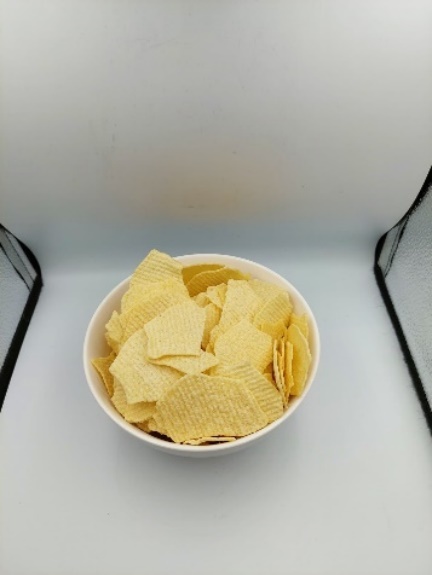 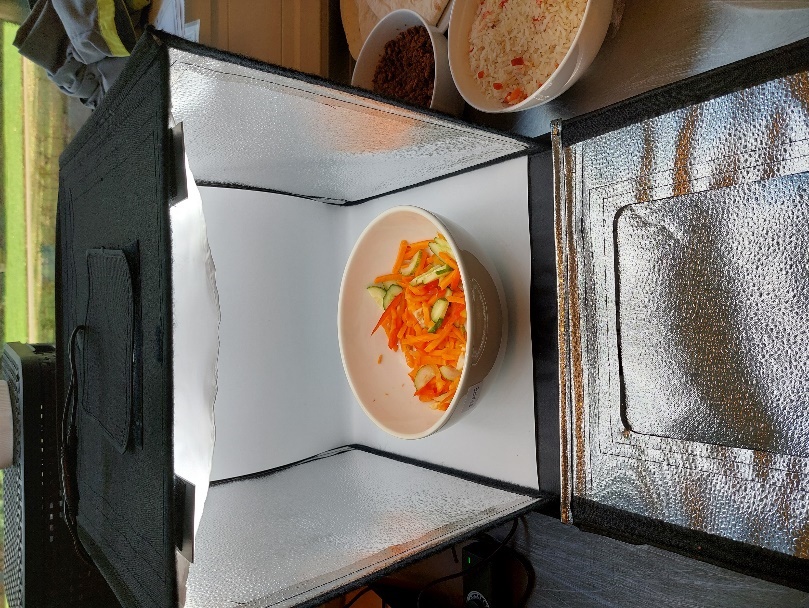 |
| *5. Hutspot*   - 1460 g Hutspot with hachee (AH private label) - 40 g Mustard (AH private label)   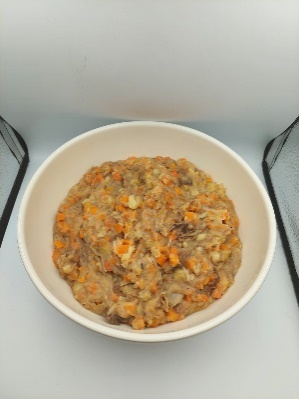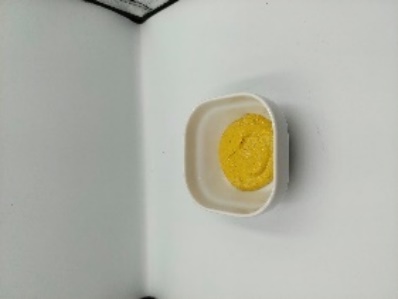 | *11. Sauerkraut dish*   - 1502 g Rösti rounds (Aviko), spiced sauerkraut (AH private label), extra lean smoked sausage (Unox) and baked onions (Go Tan)   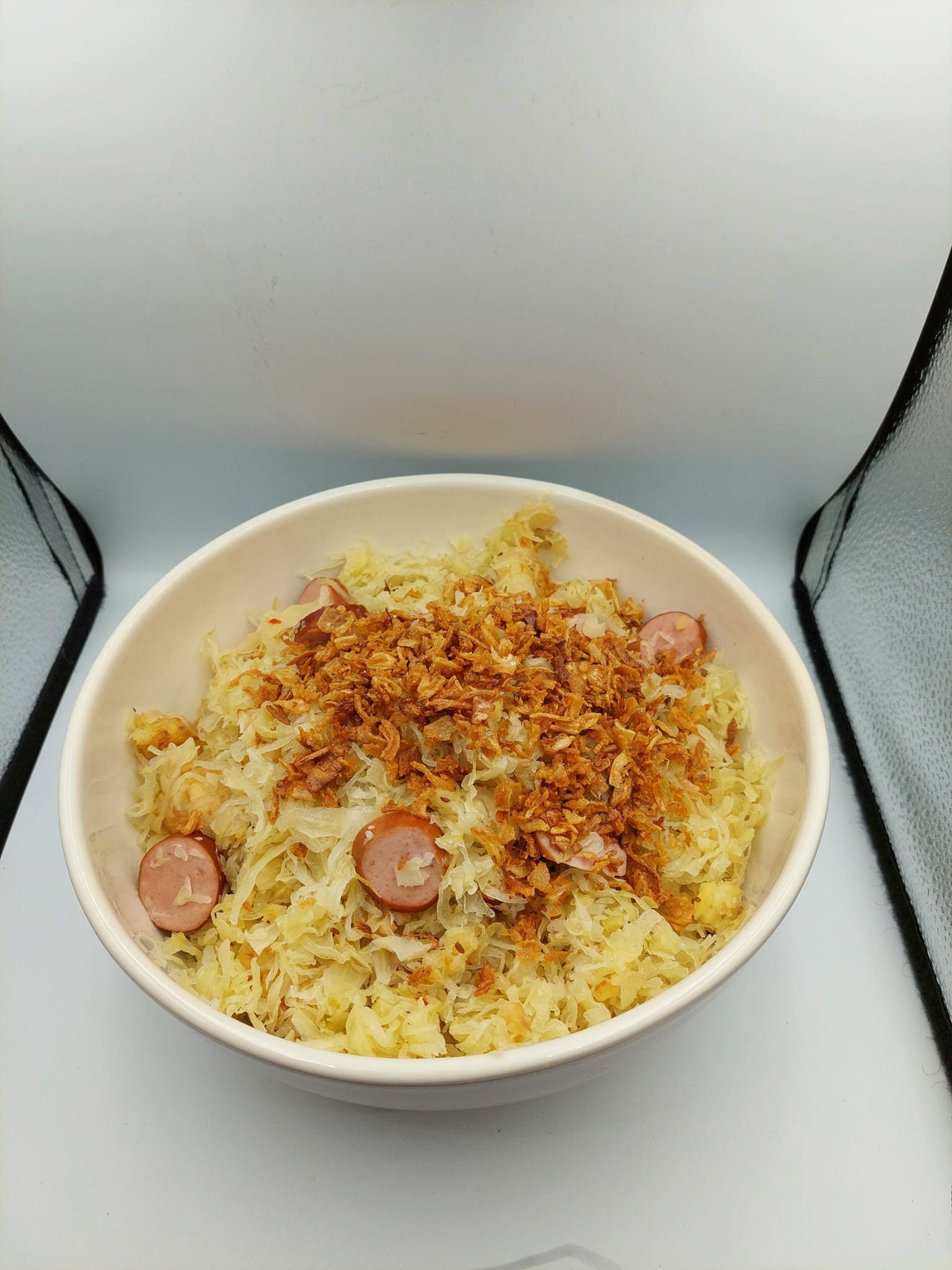 |
| *6. Quiche, mushroom soup & couscous salad*   - 575 g Quiche Lorraine (AH private label) - 348 g Mushroom soup (Unox) with liquid backing butter (Becel) - 600 g Couscous salad with pesto (AH private label)   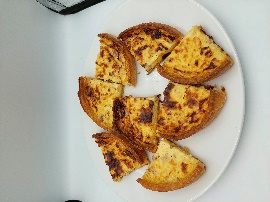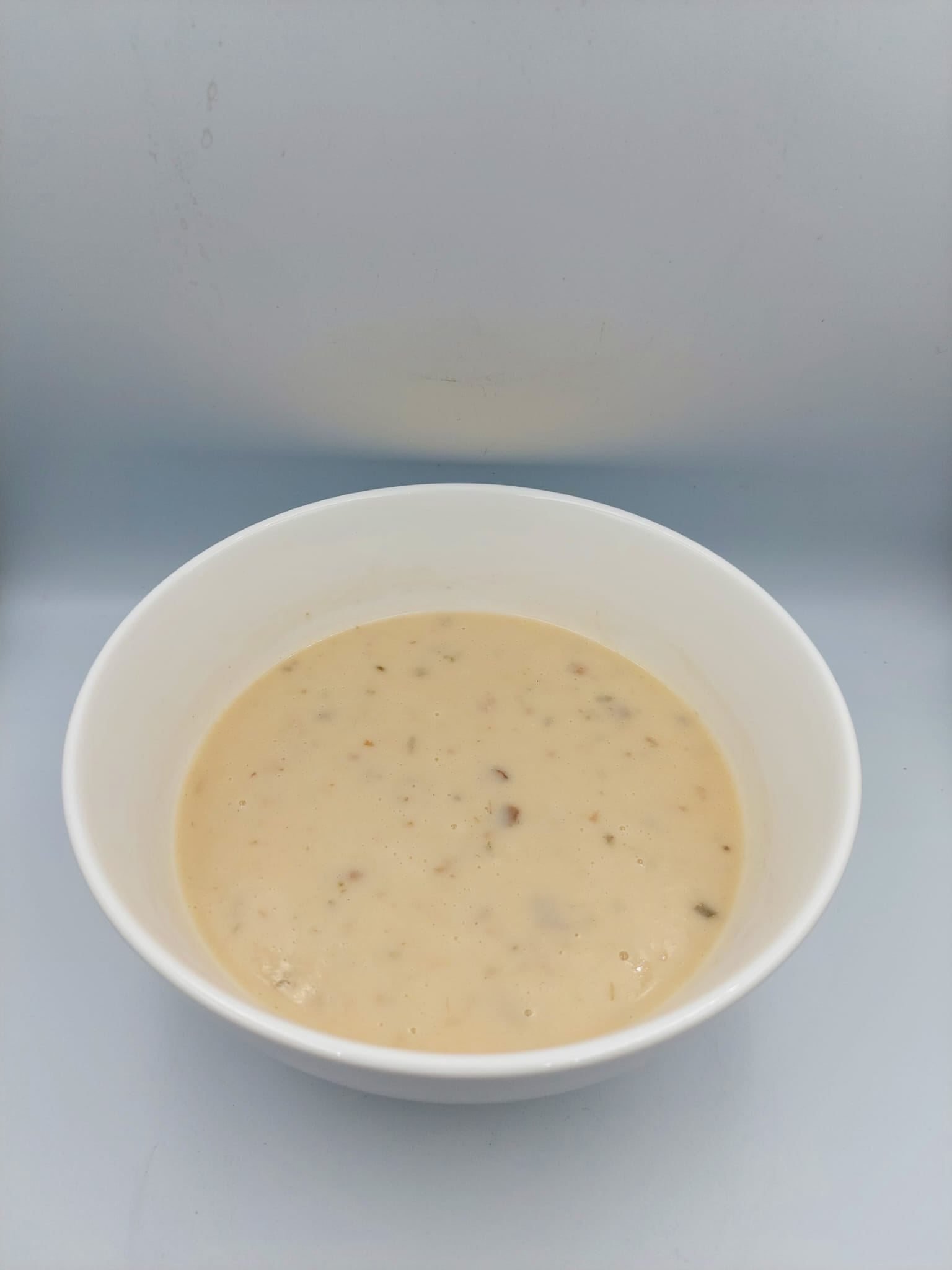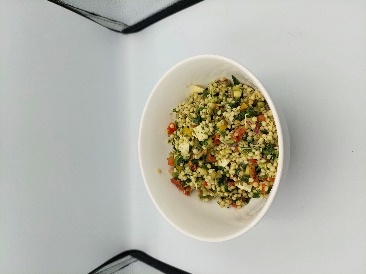 | *12. Tagliatelle, roasted bread & steamed vegetables*   - 888 g Tagliatelle fungi (Iglo) with marinated steak (AH private label) - 100 g Pan de ajo (Panetteria Di Sergio) - 516 g Farmer vegetable mix (Iglo)   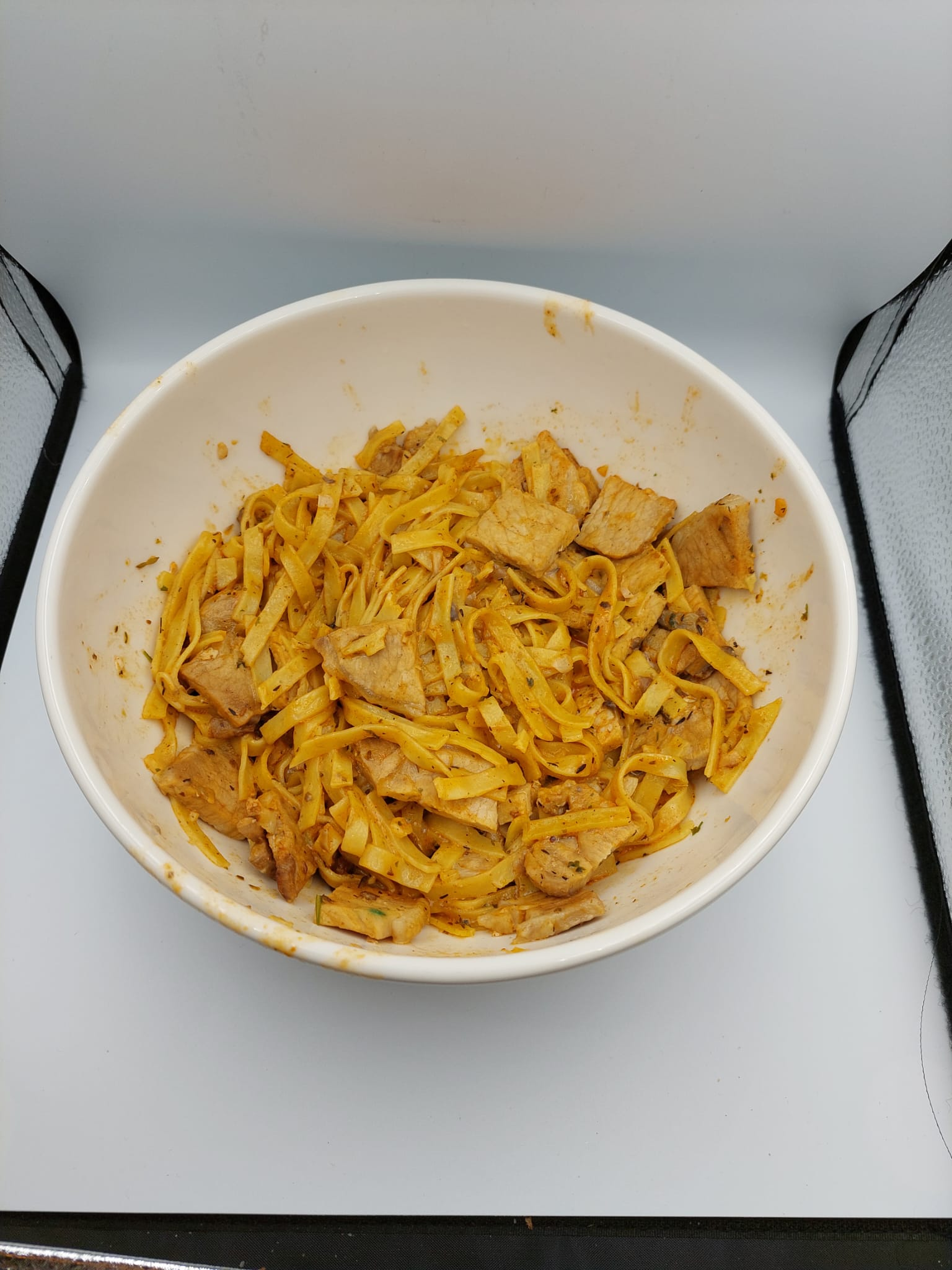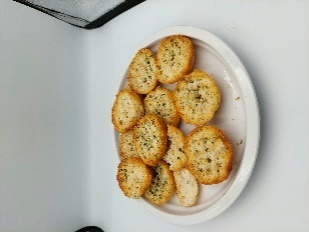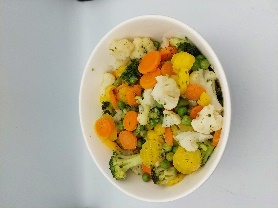 |

**Table 3.** Composition, liking and familiarity of served breakfast meals and lunch meals. Data are presented as mean ± SE.

| **Fast meals** | **Portion size (g)** | **Energy (kcal)** | **Energy density (kcal/g)** | **%EN Nova 4** | **Liking** | **Familiarity** | **Slow meals** | **Portion size (g)** | **Energy (kcal)** | **Energy density (kcal/g)** | **%EN Nova 4** | **Liking** | **Familiarity** |
| --- | --- | --- | --- | --- | --- | --- | --- | --- | --- | --- | --- | --- | --- |
| ***Breakfasts*** | |  |  |  |  |  |  |  |  |  |  |  |  |
| **FB1** | 1508 | 2719 | 1.80 | 100 | 62 ± 4 | 2.7 ± 0.5 | **SB1** | 1512 | 2751 | 1.82 | 99 | 66 ± 3 | 3.5 ± 0.4 |
| **FB2** | 1509 | 2741 | 1.82 | 100 | 73 ± 4 | 2.2 ± 0.4 | **SB2** | 1510 | 2791 | 1.85 | 100 | 63 ± 5 | 2.3 ± 0.2 |
| **FB3** | 1508 | 2559 | 1.70 | 100 | 66 ± 4 | 2.8 ± 0.4 | **SB3** | 1506 | 2563 | 1.70 | 85 | 76 ± 3 | 2.1 ± 0.2 |
| **FB4** | 1500 | 2774 | 1.85 | 78 | 61 ± 5 | 2.1 ± 0.2 | **SB4** | 1502 | 2724 | 1.81 | 78 | 49 ± 3 | 3.1 ± 0.5 |
| **FB5** | 1508 | 3468 | 2.30 | 100 | 57 ± 6 | 2.7 ± 0.4 | **SB5** | 1500 | 3441 | 2.29 | 100 | 60 ± 4 | 2.6 ± 0.5 |
| **FB6** | 1530 | 2803 | 1.83 | 100 | 61 ± 5 | 3.1 ± 0.4 | **SB6** | 1530 | 2805 | 1.83 | 90 | 77 ± 3 | 2.1 ± 0.2 |
| **Lunches** | |  |  |  |  |  |  |  |  |  |  |  |  |
| **FL1** | 1515 | 2283 | 1.51 | 98 | 66 ± 4 | 2.4 ± 0.2 | **SL1** | 1508 | 2318 | 1.54 | 56 | 57 ± 4 | 3.1 ± 0.2 |
| **FL2** | 1507 | 2440 | 1.62 | 76 | 47 ± 6 | 3.3 ± 0.5 | **SL2** | 1528 | 2732 | 1.79 | 79 | 63 ± 5 | 2.9 ± 0.4 |
| **FL3** | 1526 | 1477 | 0.97 | 85 | 54 ± 5 | 3.9 ± 0.4 | **SL3** | 1514 | 1564 | 1.03 | 83 | 62 ± 4 | 2.5 ± 0.2 |
| **FL4** | 1511 | 2119 | 1.40 | 98 | 63 ± 3 | 3.2 ± 0.2 | **SL4** | 1503 | 2913 | 1.94 | 96 | 60 ± 5 | 3.1 ± 0.4 |
| **FL5** | 1500 | 1426 | 0.95 | 97 | 51 ± 6 | 3.8 ± 0.6 | **SL5** | 1502 | 1570 | 1.04 | 89 | 41 ± 5 | 5.0 ± 0.5 |
| **FL6** | 1522 | 2940 | 1.93 | 100 | 71 ± 3 | 3.9 ± 0.4 | **SL6** | 1504 | 2517 | 1.67 | 84 | 73 ± 4 | 3.0 ± 0.2 |

FB, fast breakfasts. SB, slow breakfasts. FL, fast lunches. SL, slow lunches.

**Table 4:** Intraclass correlation coefficients (ICC) with 95% confidence interval for the oral-processing behaviours obtained with video annotation. An ICC <0.50 indicates poor consistency, 0.50-0.75 indicates moderate consistency, 0.75-0.90 indicates good consistency, and >0.90 indicates excellent consistency (Koo & Li, 2016).

| **Oral-processing behaviours** | **Breakfasts** | **Lunches** |
| --- | --- | --- |
| Bites | 0.996 (0.991; 1) | 1.000 (0.998; 1) |
| Chews | 0.999 (0.998; 1) | 1.000 (0.997; 1) |
| Sips | 1.000 (NA; NA) | 1.000 (1; 1) |
| Swallows | 0.957 (0.719; 0.995) | 0.999 (0.995; 1) |
| Meal duration | 0.990 (0.992; 1) | 1.000 (0.997; 1) |
| Meal oro-sensory exposure time | 0.920 (0.345; 0.992) | 0.998 (0.988; 1) |
| Water sip duration | 0.986 (0.887; 0.999) | 0.986 (0.868; 0.999) |

Koo, T. K., & Li, M. Y. (2016). A guideline of selecting and reporting intraclass correlation coefficients for reliability research. *Journal of chiropractic medicine, 15*(2), 155-163.

**Table 5**. Microstructure of oral processing behaviour of the individual meals. Data are presented as mean ± SE.

|  | **ED consumed (kcal/g)** | **Meal eating rate (g/min)** | **Bite size ^a^ (g)** | **Chews per bite ^a^** | **Number of chews ^a^ (g^-1^)** | **Chewing frequency ^a^ (chews/s)** | **OSE time ^a^ (min)** | **OSEg (s/g)** |
| --- | --- | --- | --- | --- | --- | --- | --- | --- |
| *Breakfasts* |  |  |  |  |  |  |  |  |
| FB1 | 1.9 ± 0.1 | 67 ± 6 | 17 ± 3 | 27 ± 4 | 1.8 ± 0.4 | 1.5 ± 0.1 | 8.1 ± 1.1 | 1.3 ± 0.3 |
| FB2 | 1.8 ± 0.1 | 67 ± 6 | 14 ± 2 | 13 ± 3 | 1.1 ± 0.3 | 1.4 ± 0.1 | 6.5 ± 0.8 | 1.0 ± 0.4 |
| FB3 | 1.4 ± 0.1 | 67 ± 6 | 17 ± 2 | 17 ± 4 | 1.2 ± 0.3 | 1.4 ± 0.1 | 6.8 ± 1.0 | 1.0 ± 0.2 |
| FB4 | 1.3 ± 0.1 | 67 ± 7 | 15 ± 2 | 16 ± 2 | 1.2 ± 0.3 | 1.5 ± 0.1 | 6.5 ± 0.6 | 0.9 ± 0.2 |
| FB5 | 2.1 ± 0.1 | 71 ± 7 | 16 ± 2 | 15 ± 1 | 1.1 ± 0.2 | 1.3 ± 0.1 | 5.3 ± 1.0 | 0.9 ± 0.2 |
| FB6 | 1.4 ± 0.1 | 101 ± 10 | 20 ± 2 | 8 ± 1 | 0.4 ± 0.1 | 1.3 ± 0.1 | 3.3 ± 0.3 | 0.4 ± 0.1 |
| SB1 | 2.0 ± 0.1 | 45 ± 3 | 11 ± 2 | 20 ± 2 | 2.0 ± 0.3 | 1.5 ± 0.1 | 8.9 ± 0.9 | 1.5 ± 0.2 |
| SB2 | 1.5 ± 0.0 | 60 ± 5 | 17 ± 2 | 20 ± 2 | 1.3 ± 0.2 | 1.4 ± 0.1 | 6.8 ± 0.6 | 1.0 ± 0.1 |
| SB3 | 1.4 ± 0.1 | 46 ± 3 | 11 ± 1 | 23 ± 4 | 2.0 ± 0.3 | 1.5 ± 0.1 | 10.9 ± 0.9 | 1.5 ± 0.2 |
| SB4 | 1.6 ± 0.1 | 62 ± 6 | 13 ± 1 | 19 ± 3 | 1.8 ± 0.5 | 1.4 ± 0.1 | 7.0 ± 0.7 | 1.3 ± 0.3 |
| SB5 | 2.3 ± 0.1 | 46 ± 3 | 9 ± 1 | 17 ± 2 | 1.9 ± 0.1 | 1.6 ± 0.1 | 7.5 ± 0.4 | 1.4 ± 0.1 |
| SB6 | 1.4 ± 0.0 | 61 ± 3 | 15 ± 1 | 18 ± 1 | 1.2 ± 0.1 | 1.4 ± 0.1 | 8.4 ± 0.8 | 1.0 ± 0.1 |
| *Lunches* |  |  |  |  |  |  |  |  |
| FL1 ^b^ | 1.5 ± 0.1 | 66 ± 5 | 10 ± 1 | 11 ± 2 | 1.1 ± 0.2 | 1.5 ± 0.1 | 8.8 ± 1.3 | 0.1 ± 0.1 |
| FL2 ^b^ | 1.6 ± 0.1 | 46 ± 3 | 10 ± 1 | 17 ± 3 | 1.7 ± 0.3 | 1.5 ± 0.1 | 7.9 ± 0.7 | 1.3 ± 0.2 |
| FL3 | 1.0 ± 0.0 | 65 ± 5 | 12 ± 2 | 13 ± 1 | 1.3 ± 0.3 | 2.0 ± 0.1 | 5.8 ± 0.6 | 1.0 ± 0.1 |
| FL4 | 1.4 ± 0.1 | 62 ± 4 | 10 ± 1 | 10 ± 1 | 1.1 ± 0.2 | 1.4 ± 0.1 | 8.1 ± 0.5 | 1.0 ± 0.2 |
| FL5 | 1.0 ± 0.0 | 79 ± 6 | 15 ± 2 | 12 ± 2 | 1.0 ± 0.3 | 1.5 ± 0.1 | 5.1 ± 0.8 | 0.9 ± 0.3 |
| FL6 | 1.9 ± 0.0 | 65 ± 5 | 11 ± 2 | 11 ± 2 | 1.1 ± 0.2 | 1.5 ± 0.1 | 6.8 ± 0.6 | 1.0 ± 0.3 |
| SL1 | 1.6 ± 0.1 | 44 ± 3 | 11 ± 1 | 16 ± 2 | 1.6 ± 0.3 | 1.5 ± 0.0 | 8.9 ± 1 | 1.3 ± 0.2 |
| SL2 ^b^ | 2.1 ± 0.1 | 37 ± 2 | 5 ± 1 | 9 ± 1 | 1.7 ± 0.3 | 1.5 ± 0.1 | 10.3 ± 0.9 | 1.6 ± 0.2 |
| SL3 | 1.3 ± 0.1 | 45 ± 3 | 9 ± 1 | 15 ± 2 | 2.1 ± 0.6 | 1.5 ± 0.1 | 9.1 ± 1.7 | 1.7 ± 0.6 |
| SL4 | 1.7 ± 0.1 | 36 ± 2 | 8 ± 1 | 15 ± 1 | 2.3 ± 0.4 | 1.5 ± 0.1 | 11.1 ± 1.2 | 1.9 ± 0.3 |
| SL5 | 1.0 ± 0.0 | 50 ± 4 | 10 ± 1 | 15 ± 2 | 1.7 ± 0.3 | 1.5 ± 0.1 | 6.5 ± 0.4 | 1.4 ± 0.3 |
| SL6 | 1.7 ± 0.1 | 53 ± 3 | 11 ± 1 | 14 ± 2 | 1.3 ± 0.1 | 1.5 ± 0.1 | 9.4 ± 0.6 | 1.0 ± 0.1 |

FB, fast breakfasts. SB, slow breakfasts. FL, fast lunches. SL, slow lunches. OSE oro-sensory exposure. ED energy density.

^a^ Assessed from video coding (n=7).

^b^ Recoding of one video not succeeded (n=6).

**Table 6.** Average liking, familiarity, expected satiation, and pre and post-appetite ratings of the fast and slow breakfast and lunch meals. Data are presented as mean ± SE.

|  | **Fast breakfasts** | **Slow breakfasts** | **P** | **Fast lunches** | **Slow lunches** | **P** |
| --- | --- | --- | --- | --- | --- | --- |
| *Pre-appetite ratings* |  |  |  |  |  |  |
| Hunger | 62 ± 2 | 61 ± 2 | 0.54 | 64 ± 7 | 66 ± 7 | 0.28 |
| Fullness | 11 ± 1 | 10 ± 1 | 0.45 | 23 ± 2 | 19 ± 2 | ***0.03*** |
| Thirst | 65 ± 2 | 64 ± 2 | 0.83 | 56 ± 6 | 56 ± 6 | 0.91 |
| Desire to eat | 52 ± 2 | 60 ± 3 | 0.42 | 66 ± 7 | 67 ± 7 | 0.45 |
| Prospective consumption | 62 ± 2 | 62 ± 2 | 0.65 | 60 ± 6 | 63 ± 7 | ***0.04*** |
| *Post-appetite ratings* |  |  |  |  |  |  |
| Hunger | 12 ± 2 | 15 ± 2 | 0.11 | 13 ± 1 | 16 ± 2 | 0.09 |
| Fullness | 71 ± 2 | 72 ± 2 | 0.73 | 73 ± 8 | 69 ± 7 | 0.11 |
| Thirst | 26 ± 2 | 38 ± 3 | ***<0.001*** | 44 ± 5 | 49 ± 5 | 0.05 |
| Desire to eat | 18 ± 2 | 18 ± 2 | 0.76 | 16 ± 2 | 17 ± 2 | 0.40 |
| Prospective consumption | 18 ± 2 | 18 ± 2 | 0.80 | 17 ± 2 | 20 ± 2 | 0.15 |

**Table 7**. Indicated reasons of participants to stop eating. ^1^Other includes: ‘I was bored with the texture’, ‘I ate the portion I would normally eat’, ‘only "unhealthy" foods are left’, ‘I want to eat the same amount as everyone else’, and ‘Enough time has passed since the start of the meal’.

| **Reason** | **Fast**  **breakfasts** | **Slow breakfasts** | **Fast lunches** | **Slow lunches** |
| --- | --- | --- | --- | --- |
| I was full | 59 (66%) | 52 (58%) | 55 (61%) | 50 (56%) |
| The food is no longer appealing to me | 13 (14%) | 9 (10%) | 14 (16%) | 11 (12%) |
| I was bored with the flavor | 5 (6%) | 5 (6%) | 9 (10%) | 10 (11%) |
| I have eaten the amount that I planned | 4 (4%) | 8 (9%) | 2 (2%) | 3 (3%) |
| Other^1^ | 9 (10%) | 16 (18%) | 10 (11%) | 16 (18%) |

**Table 8.** Beta coefficient of the repeated measures linear mixed effect model between sensory ratings of meal components and food intake (g).

|  | **β** | **P** |
| --- | --- | --- |
| Liking | 1.71 | ***<0.001*** |
| Flavour intensity | 0.27 | 0.13 |
| Hardness | -1.01 | ***<0.001*** |
| Dryness | -1.41 | ***<0.001*** |
| Thickness ^a^ | -0.16 | 0.64 |

^a^ Only for semi-solids and liquid components (n= 17 components).
